# MAML1 drives Notch and Hedgehog oncogenic pathways by inhibiting Itch activity in triple-negative breast cancer

**DOI:** 10.1038/s41418-025-01613-5

**Published:** 2025-11-21

**Authors:** Sabrina Zema, Francesca Di Fazio, Maria Pelullo, Sara Di Savino, Bruna Cerbelli, Martina Leopizzi, Laura Di Magno, Carmine Nicoletti, Giovanna Peruzzi, Daniel D’Andrea, Maria V. Giuli, Samantha Cialfi, Biagio Palmisano, Alice Turdo, Rocco Palermo, Giulia d’Amati, Gianluca Canettieri, Antongiulio Faggiano, Lucia Di Marcotullio, Matilde Todaro, Isabella Screpanti, Claudio Talora, Saula Checquolo, Diana Bellavia

**Affiliations:** 1 Department of Molecular Medicine, Sapienza University of Rome, Rome, Italy; 2 Department of Radiological, Oncological and Pathological Sciences, Sapienza University of Rome, Rome, Italy; 3 Department of Medical-Surgical Sciences and Biotechnologies, Sapienza University of Rome, Latina, Italy; 4 Department of Anatomical, Histological, Forensic Medicine and Orthopedic Sciences, Section of Histology and Embryology, Sapienza University of Rome, Rome, Italy; 5 Center for Life Nano-& Neuro-Science@Sapienza, Istituto Italiano di Tecnologia, Rome, Italy; 6 School of Engineering Mathematics and Technology, University of Bristol, Bristol, United Kingdom; 7 Institute for Complex Systems, National Research Council, Rome, Italy; 8 Department of Health Promotion Sciences, Internal Medicine and Medical Specialties, University of Palermo, Palermo, Italy; 9 Istituto Pasteur Italia, Fondazione Cenci-Bolognetti, Sapienza University of Rome, Rome, Italy; 10 Department of Clinical and Molecular Medicine, Sapienza University of Rome, Rome, Italy; 11 A.O.U.P. “Paolo Giaccone”, University of Palermo, Palermo, Italy; 12 Present Address: Casa di cura Prof. Brodetti Villa Igea, Foggia, Italy

**Keywords:** Ubiquitylation, Oncogenes

## Abstract

Triple-negative breast cancer (TNBC) is an aggressive and heterogeneous breast cancer subtype with poor patient outcomes. TNBC heterogeneity arises from multiple dysregulated pathways, including Notch and Hedgehog, which contribute to tumor initiation, progression, and drug resistance. Identifying common molecular regulators of TNBC aggressiveness is crucial for developing effective therapeutic strategies. Here, we demonstrate that the transcriptional coactivator MAML1 drives TNBC aggressiveness by regulating Notch1 and Gli1 stability through the E3 ubiquitin ligase Itch, functioning as an Itch-negative regulator. Mechanistically, MAML1 interacts with Itch *via* its PPQY motif and promotes K63-linked self-ubiquitylation of Itch, deregulating its expression/activity. Using a Maml1-deficient mouse model, we reveal an inverse correlation between MAML1 and Itch levels, where the loss of MAML1 stabilizes Itch and suppresses Notch1 and Gli1 activity. Conversely, MAML1 upregulation enhances Notch1 and Gli1 expression, driving accelerated TNBC tumor growth and faster multiorgan metastasis in vivo. Accordingly, we show that MAML1 is overexpressed in a cohort of TNBC patients, and the combined overexpression of MAML1/Notch1 and MAML1/Gli1 correlates with poor clinical outcomes by in silico analysis. Our findings establish a dual role for MAML1 as a transcriptional coactivator and a post-translational regulator of Itch, thereby amplifying Notch and Hedgehog oncogenic signaling. This study uncovers MAML1 as a key driver of TNBC progression and a potential therapeutic target for fighting TNBC aggressiveness and heterogeneity.

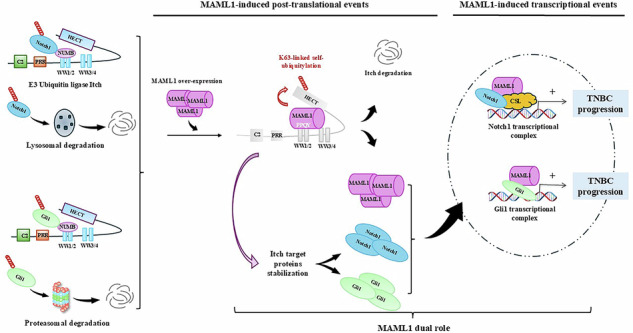

## Introduction

Breast cancer (BC) is the most frequently diagnosed tumor and the leading cause of cancer-related death [1] in women. Triple-negative breast cancer (TNBC) is the most aggressive molecular subtype of BC, associated with a high rate of metastasis [2]. Unfortunately, clinical outcomes for TNBC patients remain poor despite advances in underlying tumor biology. TNBC is recognized as a heterogeneous disease [3] that arises from various disrupted signaling pathways. Such molecular heterogeneity partly explains the tumor aggressiveness and failure of TNBC treatment. A better understanding of the molecular mechanisms underlying TNBC pathogenesis is still urgently needed to identify more precise drug targets.

Mastermind-like 1 (MAML1) was first identified as a pivotal transcriptional co-activator in the Notch pathway [4] and is now reported to be a common interactor across multiple signaling pathways [5]. MAML1 protein is characterized by two distinct transcription activation domains, TAD1 and TAD2 [6, 7]. It is well-recognized that TAD1, at the N-terminal domain, mediates the interaction of MAML1 with the Notch intracellular domain (NICD) and the CSL (CBF1/RBPjκ in mammals) transcription factor, forming the Notch-transcriptional complex to activate Notch-target genes [8]. Additionally, TAD1 is involved in the interaction with components of other pathways, namely MEF2C [9], p53 [10], and EGR1 [11]. The TAD2 is located at the C-terminal region and is essential for Notch activity in vivo [12] and interacts with components of different signaling pathways, including Wnt/β-catenin [13], Gli1, the last effector of Hedgehog (Hh) [14] and YAP in the Hippo pathway, which physically binds MAML1-PPXY motif [15]. Based on the observation that MAML1 controls numerous signaling pathways, it is not surprising that its deregulated expression has been described in several cancers [5], including TNBC [16, 17], although the underlying molecular mechanisms are poorly characterized.

Itch is an E3 ubiquitin ligase that promotes the ubiquitin-mediated proteolytic pathway of over 50 target proteins, among them components of Hedgehog [18, 19] and Notch pathways [20–23], and its dysregulation is implicated in several diseases, particularly cancer [24, 25]. Itch is a typical NEDD4 family member and shares a conserved modular architecture, with an N-terminal C2 domain, a unique proline-rich motif (PRR), four WW domains, and a C-terminal HECT domain. Itch promotes substrate poly-ubiquitylation using K48, K63, K27, K29, and K33 linkages, suggesting that it plays multiple regulatory roles via mono/poly-ubiquitylation and diverse linkages [26]. WW domains of Itch bind several proline-rich motifs, including L/PPXY motifs, facilitating the binding of Itch to specific substrates [26, 27].

Here, we reveal a non-canonical role for MAML1 as Itch-negative regulator, ultimately leading to increased stability of Itch substrates, Notch1 and Gli1, which gain oncogenic function in TNBC. Our results unveil new mechanistic insights based on the high expression levels of endogenous MAML1, which impairs the ability of Itch to recruit and ubiquitylate its substrates by inducing Itch K63-self-ubiquitylation. We demonstrate that MAML1, by combining its dual role as a transcriptional co-factor of key oncogenic pathways and a post-translational regulator of Itch target proteins, strongly supports TNBC progression. Given its multifaceted pro-tumoral role, we suggest MAML1 as a potential therapeutic target for TNBC.

## Results

### MAML1 interacts with Itch and regulates Itch-induced ubiquitylation on Gli1 and Notch1 target proteins

**Fig. 1:**
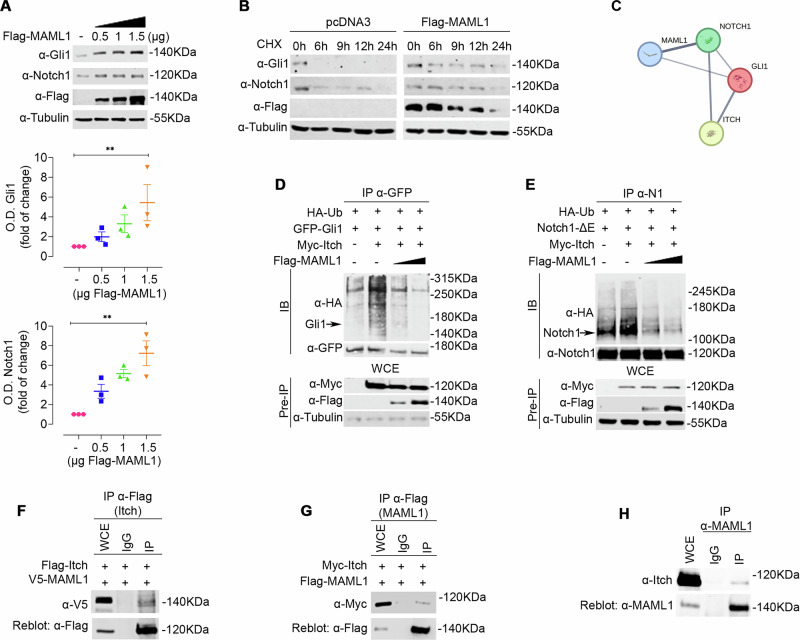
MAML1 regulates Itch-induced ubiquitin signaling on Notch1 and Gli1 by binding Itch protein. **A** Upper panel, whole-cell extracts (WCEs) of NIH3T3 after 48 h of transfection with different amounts of Flag-MAML1, as indicated in the panel. Immunoblotting (IB) of endogenous Gli1, Notch1, and Flag-tagged MAML1. Lower panel, optical densitometry (O.D.) analysis of Gli1 and Notch1 protein levels. **B** WCEs of WT MEF transiently transfected with Flag-MAML1 or pcDNA3 plasmids and treated with cycloheximide (CHX) (30μg/ml) up to 24 h to block protein synthesis. Immunoblotting analysis for Gli1, Notch1, and Flag. **C** Plot generated using STRING 9.0 (Search Tool for the Retrieval of Interacting Genes). Previously identified protein-protein interactions among MAML1, Gli1, Notch1, and Itch proteins. Black edges represent interactions; line thickness is a function of the number of previously identified interactions. In vitro ubiquitination assays of GFP-Gli1 (**D**) and Notch1-∆E, lacking the extracellular domain (**E**) from the lysates of HEK293T cells co-transfected with plasmids indicated in the panels. Gli1 immunoprecipitation (IP) with α-GFP antibody, and Notch1-∆E with α-Notch1 antibody. Immunoblotting analysis for HA-tagged ubiquitin with α-HA antibody to detect the Notch1 and Gli1 polyubiquitylated forms. The membranes were reblotted to assess the levels of immunoprecipitated protein with α-GFP and α-Notch1 antibodies, respectively. The lower panels show the immunoblots of Pre-IP WCEs by using α-Myc and α-Flag antibodies. Co-IPs of Flag-Itch and V5-MAML1 (**F**), or Flag-MAML1 and Myc-Itch (**G**) from HEK293T transfected with indicated plasmids; and Co-IP of endogenous MAML1 and Itch proteins (**H**) from WT MEFs. Densitometric analysis in panel A normalized on endogenous Tubulin; data represent Mean ± SEM of *n* = 3 independent experiments. **p < 0.01 calculated by ANOVA-Kruskal Wallis test. Representative immunoblotting of at least *n* = 3 biological replicates with similar results are shown in (**A**, **B**, **D**, **E**). Tubulin was used as the loading control. The arrows indicate the molecular weight of the immunoprecipitated protein.

Starting from the observation that increasing amounts of Flag-MAML1, transiently transfected in the NIH3T3 cell line, resulted in a dose-dependent increase in Gli1 and Notch1 protein levels (Fig. 1A), we aimed to investigate whether this effect was due to post-translational modifications that sustain protein stability. To this end, WT MEF cells were transfected with Flag-MAML1, and upon cycloheximide (CHX) treatment we observed that MAML1 ectopic expression increased the half-life of Notch1 and Gli1 proteins, compared to control cells, up to 24 h (Fig. 1B). These observations indicate that MAML1 overexpression may be involved in post-translational mechanisms that govern Notch1 and Gli1 protein stability. Notably, Itch was identified as the E3 ubiquitin ligase that drives Gli1 and Notch1 protein ubiquitylation [18–20, 22, 23]. String analysis reveals a strong interconnection between Itch, Notch1 and Gli1, but no evidence between Itch and MAML1 (Fig. 1C). To investigate whether MAML1 may counteract Itch activity, we performed ubiquitylation assays for Gli1 and Notch1 proteins in the presence of Itch. HEK293T cells were co-transfected with Myc-Itch, increasing doses of Flag-tagged MAML1, and GFP-Gli1 or Notch1-ΔE proteins, to examine the ubiquitylation status of Gli1 (Fig. 1D) and Notch1 (Fig. 1E). Remarkably, increasing doses of MAML1 show the ability to hamper Itch-induced ubiquitylation of Gli1 and Notch1 in a dose-dependent manner, suggesting its role in regulating post-translational modifications that govern Notch1 and Gli1 protein stability. These events subtend a physical association between MAML1 and Itch. To address this issue, Flag-Itch and V5-MAML1 plasmids were co-transfected into HEK293T cells, and cell extracts were subjected to co-immunoprecipitation (co-IP) assays. Figure 1F reveals a physical interaction between MAML1 and Itch proteins, confirmed by reciprocal co-immunoprecipitation (Fig. 1G) and validated by co-IP of endogenous proteins (Fig. 1H).

These data demonstrate that MAML1 and Itch can physically interact and the presence of MAML1 may counteract Itch enzymatic effects on Gli1 and Notch1 target proteins. These observations strongly suggest that MAML1 may play a role in regulating the ubiquitin signaling on Itch target proteins.

### MAML1 acts as an Itch negative regulator promoting self-ubiquitylation events via K63-linkage

**Fig. 2:**
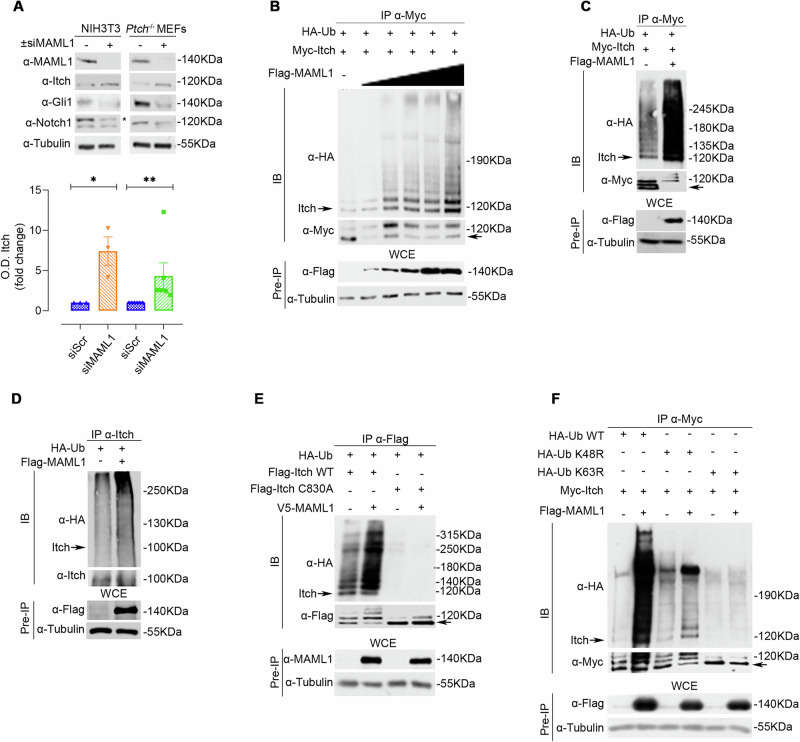
MAML1 acts as a negative regulator of Itch leading to K63-linked Itch self-ubiquitylation. **A** Representative immunoblotting of Itch, Gli1, Notch1, and MAML1 from lysate of NIH3T3 and *Ptch*^*–/–*^ MEFs upon small interference of MAML1 (siMAML1) or control cells (siScr). Lower panel, densitometric analysis of Itch protein levels: NIH3T3 7.4-fold increase; *Ptch*^*–/–*^ MEFs 4.3-fold increase. **B**,** C** In vitro ubiquitination assay, Myc-tagged Itch was immunoprecipitated with α-Myc antibody from lysates of WT MEFs co-transfected with indicated plasmids described in the panels, or in a high percentage of SDS (1%) (**C**), followed by immunoblotting with α-HA antibody to detect the Itch polyubiquitylated forms. α-Myc antibody was used to re-probe blots to assess the levels of immunoprecipitated protein. The lower panels show the immunoblots of Pre-IP WCEs by using α-Flag antibody. **D** In vitro ubiquitination assay of endogenous Itch from lysates of WT MEFs, transfected as described in the figure. Immunoprecipitation of Itch with α-Itch antibody and immunoblotting for α-HA antibody to detect the Itch polyubiquitylated forms. α-Itch antibody was used to re-probe blots to assess the levels of immunoprecipitated protein. The lower panels show the immunoblots of Pre-IP WCEs by using α-Flag antibody. **E** MAML1 induces Itch self-ubiquitination in WT MEFs transfected with indicated plasmids described in the panel. The cell lysates were immunoprecipitated with α-Flag antibody, immunoblotted for α-HA to detect the Itch polyubiquitylated forms. α-Flag antibody was used to re-probe blots to assess the levels of immunoprecipitated protein. The lower panels show the immunoblots of Pre-IP WCEs by using α-MAML1 antibody. **F** In vitro ubiquitination assay of Myc-Itch from lysates of WT MEFs transfected as indicated. Proteins were immunoprecipitated with α-Myc antibody and immunoblotted with α-HA antibody to detect the Itch polyubiquitylated forms. α-Flag antibody was used to re-probe blots to assess the levels of immunoprecipitated protein. The lower panels show the immunoblots of Pre-IP WCEs by using α-Flag antibody. Densitometric analysis in panel A normalized on endogenous Tubulin; data represent Mean ± SEM of *n* = 3 (NIH3T3) and *n* = 6 (*Ptch*^*–/–*^ MEF) independent experiments; *p < 0.05, **p < 0.01 calculated by two-tailed ratio paired *t* test. Representative immunoblotting of at least *n* = 3 biological replicates with similar results are shown in **B**–**F**. Tubulin was used as the loading control. The arrows indicate the molecular weight of the immunoprecipitated protein.

**Fig. 3:**
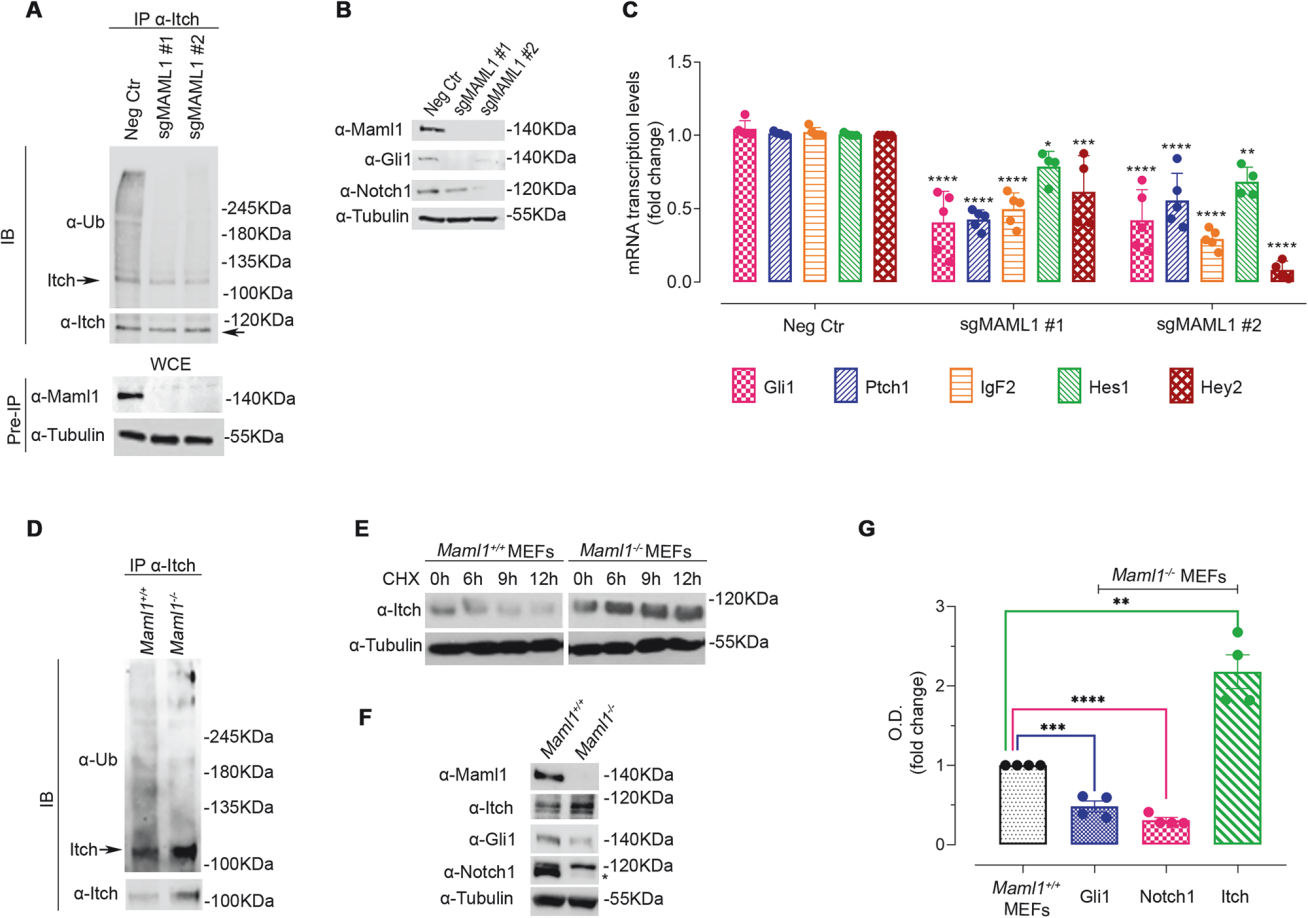
MAML1 knock-out decreases Itch ubiquitination resulting in a drastic reduction of Gli1 and Notch1 target proteins. **A** In vitro ubiquitination assay in WT MEFs gene-edited for MAML1 knock-out (sg MAML1#1 and #2), compared to the control (Neg Ctr). Endogenous Itch was immunoprecipitated with α-Itch antibody, then immunoblotted with α-Ub to detect the Itch polyubiquitylated forms. α-Itch antibody was used to re-probe blots to assess the levels of immunoprecipitated protein. The lower panels show the immunoblots of Pre-IP WCEs by using α-MAML1 antibody. **B** WCEs from (**A**) were subjected to immunoblotting analysis using α-Gli1, α-Notch1 and α-MAML1 antibodies, compared to the control. **C** Relative mRNA levels of genes regulated by Gli1 (*Gli1, Ptch1, IgF2*), and Notch1 (*Hes1* and *Hey2*) from WT MEFs gene-edited for MAML1 (sg MAML1#1 and #2), compared to the control (Neg Ctr), by qRT-PCR. Data are normalized to endogenous *Hprt* expression levels, expressed as the fold change (FC) versus the control sample value. **D** Ex vivo ubiquitination assay of Itch in MEFs derived from *Maml1*^*–/–*^ and *Maml1*^*+/+*^ mouse models. IP performed with α-Itch antibody and immunoblotting with α-Ubiquitin to detect the endogenous Itch polyubiquitylated forms. α-Itch antibody was used to re-probe blots to assess the levels of immunoprecipitated protein. **E** Representative immunoblots of Itch in cycloheximide assay in WCE from *Maml1*^*–/–*^ and *Maml1*^*+/+*^ MEFs along a time course, as indicated in the panel, to block protein synthesis. **F** Representative immunoblots for Itch, Gli1, Notch1, and MAML1 in WCE from *Maml1*^*–/–*^ and *Maml1*^*+/+*^ MEFs and panel **G** densitometric analysis of Gli1, Notch1 and Itch, relative to panel **F**, normalized on endogenous Tubulin. Panel **C** represents Mean of ± SEM of *n* = 5 (*Gli1, Ptch1, IgF2*), *n* = 4 independent experiments (*Hes1 and Hey2)*; *p < 0.05, **p < 0.01; ***p < 0.001; ****p < 0.0001 calculated by 2wayANOVA. Panel F represents Mean of ± SEM of *n* = 4 independent experiments, **p < 0.01; ***p < 0.001; ****p < 0.0001 calculated by two-tailed unpaired *t *test. Representative immunoblotting of at least *n* = 3 biological replicates with similar results are shown in **B**, **E**–**F**. Tubulin was used as the loading control. The arrows indicate the molecular weight of the immunoprecipitated protein.

Given the observation that MAML1 and Itch physically interact, we hypothesized a role for MAML1 in directly controlling Itch expression/activity, resulting in post-translational regulation of Itch target proteins. To this aim, MAML1 was knocked down with MAML1-small interference RNA (siRNA MAML1) in NIH3T3 and *Ptch*^*–/–*^ MEF cells [14]. In Fig. 2A, immunoblot analysis shows an up-regulation of endogenous levels of Itch protein, as confirmed by optical densitometry (O.D.) analysis (bottom panel), linked to a significant downregulation of Gli1 and Notch1 proteins. These data strongly suggest that MAML1 may regulate Itch expression. To investigate MAML1 ability to control Itch ubiquitylation, we performed a ubiquitylation assay in WT MEFs, transiently transfected with HA-Ub, Myc-Itch, and increasing doses of Flag-MAML1. Figure 2B reveals that MAML1 triggers Itch ubiquitylation in a dose-dependent manner, also confirmed by ubiquitylation assays in the presence of a high percentage of SDS (1%) to dissociate Itch-interacting proteins (Fig. 2C). Accordingly, MAML1 ectopic expression induces a significant increase in endogenous Itch ubiquitylation in WT MEFs (Fig. 2D). This evidence strongly suggests a direct role for MAML1 in regulating Itch ubiquitylation events. Notably, it is known that Itch undergoes a self-ubiquitylation process to regulate its activity on target proteins [28, 29]. To gain insight into the mechanism by which MAML1 regulates Itch ubiquitylation/stability, we carried out a ubiquitylation assay in WT MEFs, transfected with plasmid vectors encoding for Flag-Itch wild type (Flag-Itch WT) or dead mutant C830A (Flag-Itch-C830A) that is characterized by a mutation in the cysteine reactive site in the HECT domain. Figure 2E shows that MAML1 is unable to restore Itch ubiquitylation levels of the vector Flag-Itch-C830A, compared to the control, demonstrating that MAML1 can trigger Itch self-ubiquitylation events. To analyze the nature of ubiquitin attachment and the type of linkage forming the poly-Ub chain, we performed ubiquitylation assays using ubiquitin wild-type (HA-Ub WT) or mutant ubiquitin vectors, HA-Ub K48R or HA-Ub K63R [30]. Figure 2F shows that MAML1 is unable to induce Itch self-ubiquitylation events in the presence of the HA-Ub-K63R mutant vector, suggesting that the K63-linkage governs the process. To further investigate the role of MAML1 in regulating Itch expression and its activity on target proteins, we used CRISPR/Cas9 technology to deplete Maml1 in the WT MEF cell line (sgMAML1#1 and sgMAML1#2 clones). Endogenous ubiquitylation levels of Itch are strongly decreased in the absence of Maml1 by immunoprecipitation assays (Fig. 3A), reflecting higher enzyme activity on target proteins, as highlighted by lower expression levels of Gli1 and Notch1 proteins, than the control (Fig. 3B). Significantly, Gli1 and Notch1 downregulation impaired their transcriptional activity, with a sharp decrease in the expression levels of Gli1 (*Gli1, Ptch1, IgF2*) [14] and Notch1 (*Hes1, Hey2*) [8, 14] target genes, revealed by qRT-PCR assays (Fig. 3C). To validate these findings, we used MEF cells derived from the mouse model depleted of Maml1 (*Maml1*^*–/–*^) [14, 31] and control mice (*Maml1*^*+/+*^), and we investigated Itch ubiquitylation status. Significantly, we observed elevated levels of the immunoprecipitated Itch protein in *Maml1*^*-/-*^ MEFs compared to *Maml1*^*+/+*^ MEFs, as a result of reduced levels of protein ubiquitylation (Fig. 3D), corresponding to its greater stabilization, as confirmed upon CHX treatment (Fig. 3E). This observation suggests that Itch is protected from MAML1-induced degradation mechanisms in *Maml1*^*–/–*^ MEFs. Accordingly, Itch increased expression shows higher enzymatic activity, as revealed by the drastic reduction of Gli1 and Notch1 target proteins in *Maml1*^*–/–*^ MEF, compared to *Maml1*^*+/+*^ MEF cells (Fig. 3F, G).

Our results unveil a new function of MAML1 as a negative regulator of Itch, triggering self-ubiquitylation events via K63-linkage, resulting in strong up-regulation of Notch1 and Gli1 target proteins.

### MAML1-PPQY/Itch-WW motifs interaction triggers MAML1-dependent Itch self-ubiquitination

**Fig. 4:**
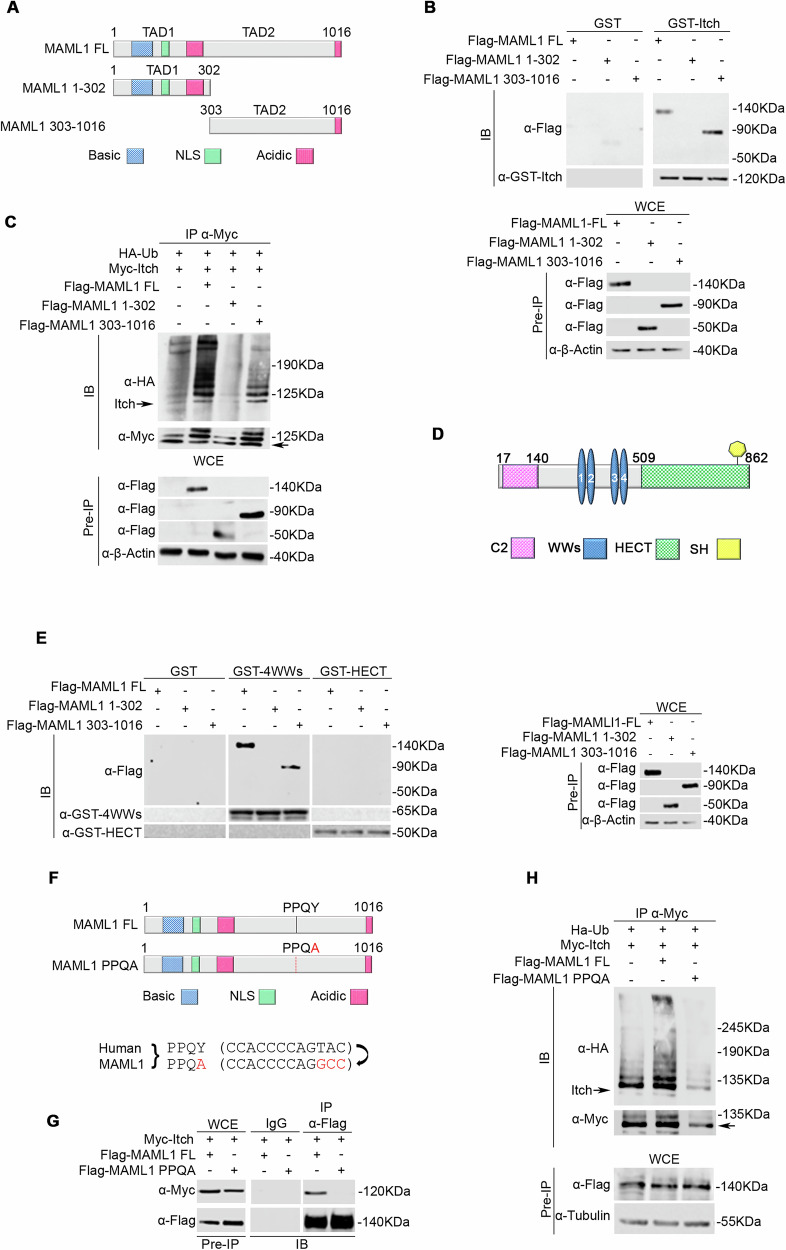
MAML1 binds Itch through the PPQA motif located at the C-terminal domain. **A** Schematic diagram of MAML1 full-length (FL) structure and its mutants by deletion (MAML1 1-302; Maml1 303-1016). **B** In vitro binding assay of recombinant GST-tagged Itch and Flag-tagged MAML1 FL, Flag-MAML1 1-302 and Flag-MAML1 303-1016. Immunoblot analysis was performed with α-Flag antibody. The membrane was reblotted with an α-GST antibody. The bottom panel shows the Pre-IP WCE by using α-Flag antibody. **C** In vitro ubiquitination assays in WT MEFs transfected with indicated plasmids. Immunoprecipitation of Itch with α-Myc antibody and immunoblotting of HA-tagged Ubiquitin by α-HA antibody to detect the Itch polyubiquitylated forms. α-Myc antibody was used to re-probe blots to assess the levels of immunoprecipitated protein. The lower panel shows the Pre-IP WCEs by using α-Flag antibody. **D** Schematic diagram of Itch structure. **E** In vitro binding assay of recombinant Itch domains (GST-4WWs and GST-HECT) with Flag-MAML1 FL, Flag-MAML1 1-302, and Flag-MAML1 303-1016. Immunoblot analysis was performed with α-Flag antibody. The membrane was reblotted with α-GST antibody. The right panel shows the Pre-IP WCEs by using α-Flag antibody. **F** Schematic diagram of Maml1 PPQY motif and the single point mutation PPQA. **G** Co-immunoprecipitation analysis followed by Immunoblotting from WCEs of HEK293T cells co-transfected with the plasmids indicated in the panel. Immunoprecipitation was performed with α-Flag antibody and immunoblotting with α-Myc antibody. The membrane was reblotted with an α-Flag antibody. **H** In vitro ubiquitination assay of Myc-Itch from WT MEFs co-transfected with Flag-MAML1 FL or Flag-MAML1 PPQA and HA-Ubiquitin. Immunoprecipitation with α-Myc antibody and immunoblotting with α-HA antibody. The membrane was reblotted with α-Myc antibody. The lower panel shows the Pre-IP WCE by using α-Flag antibody. Representative immunoblotting of at least *n* = 3 biological replicates with similar results are shown in (**B**, **C**, **E**, **G**, **H**). Tubulin and β-actin were used as the loading control. The arrows indicate the molecular weight of the immunoprecipitated protein.

To further investigate the MAML1 domain involved in the functional interaction with Itch, HEK293T cells were transiently transfected with Flag-MAML1-FL or Flag-MAML1 1-302 or Flag-MAML1 303-1016, corresponding respectively to TAD1 N-terminal and TAD2 C-terminal domains, as represented in Fig. 4A [14]. Figure 4B highlights a direct interaction between GST-tagged Itch protein and MAML1 C-terminal domain by in vitro binding assays. To gain insight into the molecular mechanisms underlying the MAML1 role in regulating Itch expression/activity, we investigated Itch ubiquitylation status in WT MEF cells, in the presence of MAML1-FL or MAML1 mutant vectors. Unlike the N-terminal domain, the C-terminal domain of MAML1 is required to induce Itch self-ubiquitylation (Fig. 4C). Next, to assess the specific region of Itch able to interact with MAML1, we performed in vitro binding assays using purified proteins corresponding to Itch functional domains, WWs, and HECT (Fig. 4D). Flag-MAML1-FL or Flag-MAML1 mutant proteins were transfected into HEK293T cells and subjected to GST pull-down assays with recombinant GST-4WWs or GST-HECT domains. Figure 4E reveals that MAML1 C-terminal domain directly interacts with Itch WWs domain. Interestingly, the MAML1 TAD2 sequence presents a PPQY motif, recognized as a binding sequence by the WWs motif [15]. To test whether PPQY motif mediates the interaction between MAML1 and Itch, we generated a Flag-tagged MAML1-FL vector with a single mutation in the PPQY sequence (Flag-MAML1-PPQA), as illustrated in Fig. 4F. HEK293T cells were co-transfected with Myc-Itch and Flag-MAML1-FL or Flag-MAML1-PPQA to perform co-immunoprecipitation assays. Figure 4G shows that the single-point mutation in the PPQY sequence was sufficient to abrogate the interaction between MAML1 and Itch WWs domain (Fig. 4G) and determined the loss-of-function of MAML1 in controlling Itch self-ubiquitylation (Fig. 4H).

These results demonstrate that MAML1 and Itch interact through PPQY/WWs motifs, and this interaction is necessary to induce MAML1-dependent Itch autocatalytic activity.

### Increased MAML1 expression promotes TNBC malignancy by switching off Itch activity on oncogenic targets Notch1 and Gli1

**Fig. 5:**
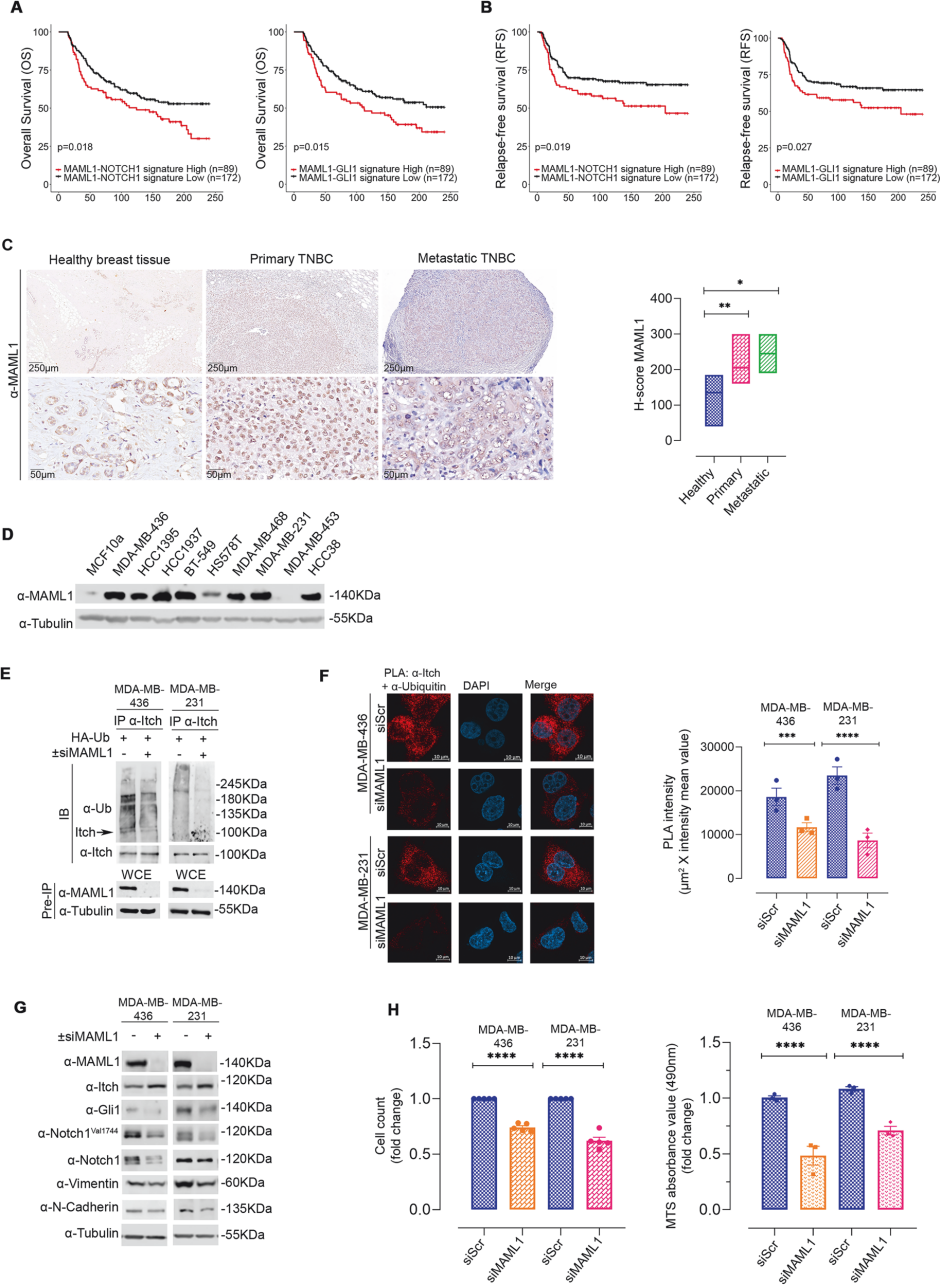
Reverse correlation between MAML1 and Itch in TNBC. Overall Survival (OS) (**A**) and Relapse-Free Survival (RFS) (**B**) Kaplan-Meier curves in Triple Negative Breast Invasive Ductal Carcinoma patients (*n* = 261) from METABRIC database. Patients were stratified in two groups, High and Low MAML1/Notch1 (left panel) and MAML1/Gli1 (right panel) signature, based on the expression levels of the MAML1/Notch1 and MAML1/Gli1 signature, using the higher tertile as threshold. Statistical significance was assessed using the log-rank test. The *p*values and the number of patients in each group are shown. **C** Left panel: representative immunohistochemical staining (IHC) of MAML1 expression in primary and metastatic human TNBC, compared to healthy tissue (scale bar: 250 μm or 50 μm, as indicated in the panels). Immunohistochemistry for MAML1 shows different intensities of staining in human TNBC. Right panel: H-scores analysis for MAML1 immunohistochemical staining by one-tailed unpaired *t* test; *p < 0.05, **p < 0.01. **D** Representative immunoblot analysis of MAML1 in different TNBC cell lines, compared to non-tumorigenic cell line MCF10a. **E** In vitro ubiquitination assay of endogenous Itch in MDA-MB-436 and MDA-MB-231, upon small interference of MAML1 (±siMAML1). Immunoprecipitation of Itch with α-Itch antibody and immunoblotting for Ubiquitin to detect the Itch polyubiquitylated forms. α-Itch antibody was used to re-probe blots to assess the levels of immunoprecipitated protein. The lower panel shows the Pre-IP WCE by using α-MAML1 antibody. **F** PLA analysis by confocal microscope of MDA-MB-436 and MDA-MB-231, upon 48 h of MAML1 silencing (siMAML1), compared to control cells (siScr). Itch ubiquitination levels were detected using a couple of probes α-Itch antibody and α-Ub antibody. Red fluorescence indicates the endogenous levels of Itch ubiquitination. Cell nuclei were stained with DAPI. The right panels show the data, indicated as indicated as intensity mean value (scale bar: 10 μm). **G** Representative immunoblots for MAML1, Itch, Gli1, Notch1^Val1744^, Notch1, Vimentin, N-Cadherin, and PCNA of MDA-MB-436 and MDA-MB-231 upon small interference of MAML1 (±siMAML1). **H** Left panel: analysis of cell counts in MDA-MB-436 and MDA-MB-231, upon MAML1 silencing (siMAML1), compared to control cells (siScr). Right panel: analysis of MTS absorbance value (490 nm) in MDA-MB-436 and MDA-MB-231, upon MAML1 silencing (siMAML1), compared to control cells (siScr). Data in panel (**F** and **H**) represent Mean ± SEM of *n* = 3 (panel **F** and left panel **H**) and *n* = 5 (right panel **H**) i*n*dependent experiments; ***p < 0.001; ****p < 0.0001 calculated by two-tailed unpaired *t* test. Representative immunoblotting of *n* = 3 biological replicates with similar results are shown in (**D**, **E**, and **G**). Tubulin was used as loading control. The arrows indicate the molecular weight of the immunoprecipitated protein.

MAML1 overexpression has been reported to sustain tumorigenesis processes in several tumor types [32–37], and is highly correlated with unfavorable clinical outcomes for patients with BC [16], including TNBC [16, 17], but the underlying molecular mechanisms are poorly characterized. Interestingly, higher expression levels of Notch1 [38] and Gli1 [39] have been described in TNBC, compared to other BC subtypes. Here, we show a MAML1-Notch1 or MAML1-Gli1 positive correlation by in silico analysis in TNBC patients (Supplementary Fig. 1A, B). Next, we evaluated the association between co-expression patterns of MAML1- Notch1 and MAML1- Gli1 with clinical outcomes in TNBC using the METABRIC dataset. Interestingly, Fig. 5A, B shows a significant association between shorter overall survival (OS) and relapse-free survival (RFS) in TNBC patients with elevated MAML1/Notch1 and MAML1/Gli1 gene signature expression, when analyzed in combination rather than individually (Supplementary Fig. 2A, B). We performed MAML1 immunohistochemical (IHC) staining on 11 surgical samples derived from patients with TNBC not treated with neoadjuvant therapy, and 5 patients without breast neoplasm undergoing esthetic plastic surgery (Table 1). Immunohistochemical analysis in Fig. 5C reveals higher levels of MAML1 in neoplastic cells, both in primary and metastatic tissue, compared to healthy tissue. Interestingly, patients with larger tumors (pT2) and patients with lymph node involvement (pN1) or distal metastasis were characterized by higher expression of MAML1, suggesting its possible role in TNBC progression (Table 1).

**Table 1:** Clinico-pathological features of the study populations.

**Sex**		
female	11	100%
male	0	0%
**Tumor histotype**		
Invasive no special type carcinoma	11	100%
**Tumoral grading**		
G1	0	0%
G2	0	0%
G3	11	100%

			MAML-1					
			low		intermediate		high	
**Age**								
<=60	5	45%	0	0%	3	27%	2	18%
>60	6	55%	0	0%	2	18%	4	36%
**T Classification**								
pT1	7	64%	0	0%	4	36%	3	27%
pT2	3	27%	0	0%	0	0%	3	27%
pT3	1	9%	0	0%	1	9%	0	0%
pT4	0	0%	0	0%	0	0%	0	0%
**N Classification**								
N0	10	91%	0	0%	5	45%	5	45%
N1	1	9%	0	0%	0	0%	1	9%
N2	0	0%	0	0%	0	0%	0	0%
N3	0	0%	0	0%	0	0%	0	0%
**M Classification**								
M0	10	91%	0	0%	5	45%	5	45%
M1	1	9%	0	0%	0	0%	1	9%
**Breast tissue from healthy patients**			2	20%	3	30%	0	0%

**Fig. 6:**
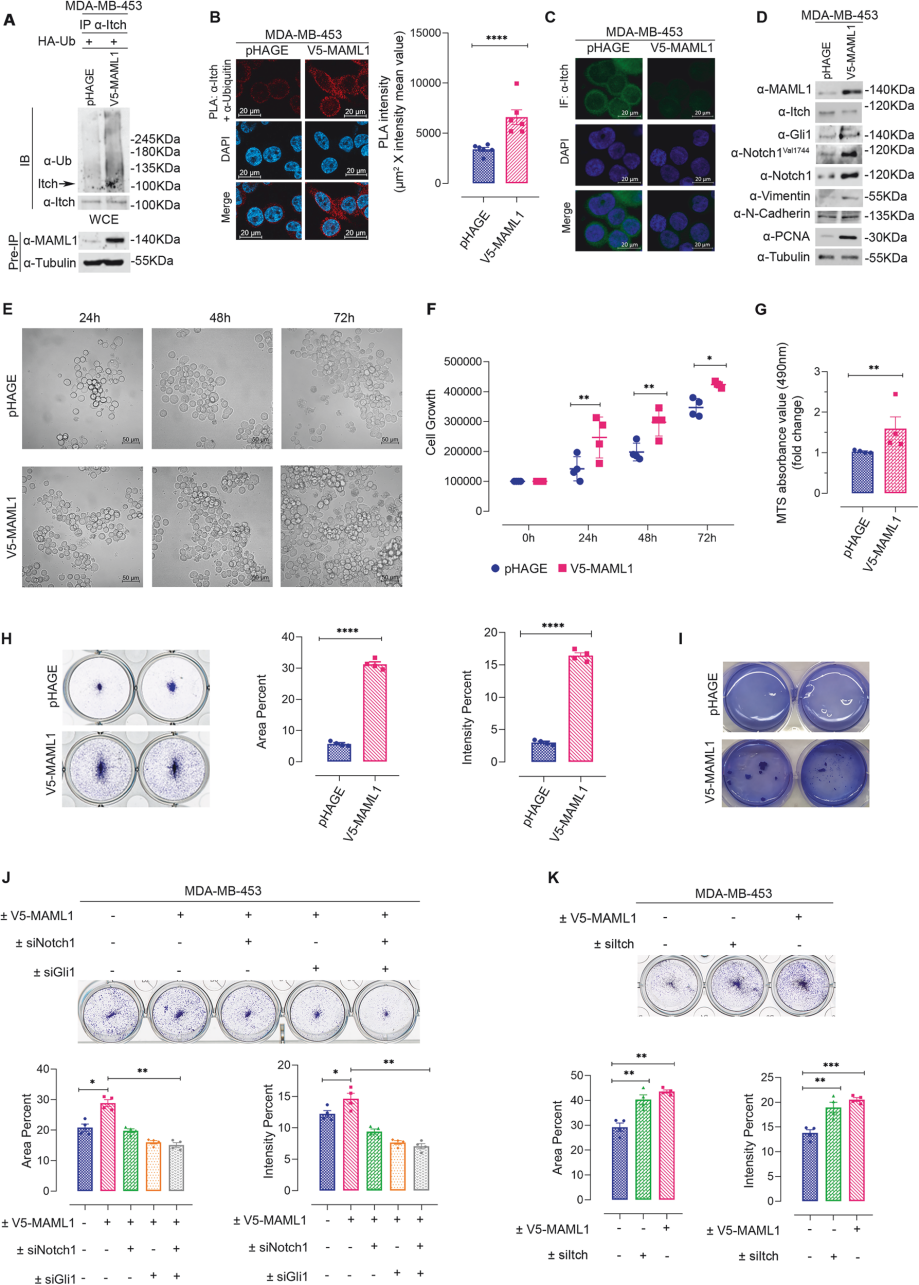
MAML1 overexpression sustains TNBC aggressiveness by hampering Itch activity. **A** MAML1 overexpression sustains Itch ubiquitination in in vitro ubiquitination assay of endogenous Itch in MAML1 stably transfected MDA-MB-453. Immunoprecipitation of Itch was performed with α-Itch antibody and immunoblotting for Ubiquitin to detect the Itch polyubiquitylated forms. The membrane was reblotted with α-Itch antibody to assess the levels of immunoprecipitated protein. The lower panel shows the Pre-IP WCE by using α-MAML1 antibody. **B** Confocal microscope PLA analysis of MB-453-V5-MAML1, compared to MB-453-pHAGE control cells. Itch ubiquitination levels were detected using α-Itch antibody and α-Ub antibody. Red fluorescence indicates the endogenous Itch protein ubiquitination. Cell nuclei were stained with DAPI. The right panel shows the data, indicated as intensity mean value (scale bar: 20 μm). **C** Confocal microscope immunofluorescence analysis of Itch protein levels in MDA-MB-453-V5-MAML1, compared to control cells. α-Itch antibody was revealed by Alexa Fluor antibody. Cell nuclei were stained with DAPI. Representative pictures of *n* = 3 independent experiments with similar results (scale bar: 20 μm). **D** Representative immunoblot assays for Itch, Gli1, Notch1^Val1744^, Notch1, Vimentin, N-Cadherin, PCNA, in MB-453-V5-MAML1 compared to control cells. Representative picture (**E**) (scale bar: 50 µm) and analysis cell count (**F**) of MDA-MB-453-V5-MAML1 cell growth, compared to control (MDA-MB-453-pHAGE), along a time course as indicated in the panel. **G** Analysis of MTS absorbance value (490 nm) in MDA-MB-453-V5- MAML1, compared to control (MDA-MB-453-pHAGE). **H** Long-term survival analyzed by a colony-forming assay of MB-453-V5-MAML1 and control cells. Left panel shows the surface coverage by the colonies represented as Area percent; right panel shows the number of colonies as Intensity percent. **I** Soft agar colony formation assays to analyze the clonogenic capacity of MDA-MB-453-V5- MAML1, compared to control. **J** Long-term survival analyzed by a colony-forming assay of MB-453-V5-MAML1 upon silencing of Notch1 and Gli1, alone and in combination, as indicated in the upper panel. The analysis was performed against control cells (MB-453-V5-MAML1 siScr; MB-453-pHAGE siScr). Bottom panel shows the surface coverage by the colonies represented as Area percent and the number of colonies as Intensity percent. **K** Long-term survival analyzed by a colony-forming assay of MB-453-V5-pHAGE upon Itch silencing and control cells (MB-453-pHAGE and MB-453-V5-MAML1 transfected with siScr). Bottom panel shows the surface coverage by the colonies represented as Area percent and the number of colonies as Intensity percent. Panel **B** shows the data of *n* = 6 independent experiments represented as Mean ± SEM, ****p < 0.0001 calculated by two-tailed unpaired t-test. Panel **F** show *n* = 4 indepe*n*dent experiments, data represent Mean ± SD; *p < 0.05; **p < 0.01 calculated by 2way ANOVA test. Panel **G** show *n* = 4 independent experiments, data, indicated as fold change, represent Mean ± SEM; **p < 0.01 calculated by two-tailed ratio paired *t* test. Panel **H** represent Mean ± SEM of *n* = 4 independent experiments; ****p < 0.0001 calculated by two-tailed unpaired *t* test. Statistical analysis of panel **J** and **K** versus siScr pHAGE or siScr V5-MAML1 calculated by two-tailed unpaired *t* test. *p < 0.05; **p < 0.01; ***p < 0.001. Representative immunoblotting of *n* = 3 biological replicates with similar results are shown in (**A** and **D**). The pictures are representative of *n* = 4 independent experiments. Tubulin was used as loading control. The arrows indicate the molecular weight of the immunoprecipitated protein.

To evaluate the MAML1 antagonist role against Itch in TNBC, we first analyzed MAML1 expression levels in several TNBC cell lines. Figure 5D shows that MAML1 is strongly up-regulated in most TNBC cell lines, compared to non-tumorigenic cells (MCF10a). To investigate whether MAML1 overexpression was responsible for regulating Itch expression, we performed MAML1 silencing by small interference RNA (siMAML1) in MDA-MB-436 and MDA-MB-231 cell lines. Consistent with our hypothesis, ubiquitylation assays demonstrated that endogenous Itch was significantly less self-ubiquitinated upon MAML1 silencing, compared to control cells (Fig. 5E), also visualized by in-situ proximity ligation assay (PLA) (Fig. 5F). Interestingly, in MAML1-depleted TNBC cell lines the absence of MAML1 greatly reduced the level of Itch self-ubiquitylation, resulting in a significant increase of endogenous Itch expression levels and its catalytic activity, as shown by drastic downregulation of Itch target proteins, Gli1 and Notch1^Val1744^ (Fig. 5G). These events trigger a down-regulation of Notch and Hh target proteins, as Vimentin and N-Cadherin [40, 41] (Fig. 5G), and a significant decrease in cell proliferation (Fig. 5H). Conversely, to investigate the MAML1 role, we generated MDA-MB-453-V5-MAML1 cells by stably transfecting ectopic MAML1 in MDA-MB-453 cells, expressing low levels of endogenous MAML1 (Fig. 5D). MAML1 overexpression triggered strong endogenous Itch ubiquitylation, compared to control cells (MDA-MB-453-pHAGE), as demonstrated through ubiquitylation assays, by immunoblotting (Fig. 6A) or revealed by PLA assays (Fig. 6B). Furthermore, MAML1 ectopic expression in MDA-MB-453-V5-MAML1 cells was associated with a drastic down-regulation of endogenous Itch, as revealed by immunofluorescence (Fig. 6C) and immunoblotting assays (Fig. 6D), and a decrease in Itch catalytic activity, as evidenced by increased expression levels of Itch target proteins, Gli1 and Notch1^Val1744^ (Fig. 6D). Significantly, the enforced levels of MAML1 ultimately empowered Notch and Hh pathways, inducing the activation of target proteins, as PCNA, α-Vimentin and N-Cadherin [14, 40, 41], directly involved in tumor progression (Fig. 6D). Accordingly, MDA-MB-453-V5-MAML1 cells showed a significant increase in cellular growth (Fig. 6E–G, Supplementary Fig. 3), long-term survival, measured in colony-forming assays (Fig. 6H), and an enhanced clonogenic capacity in anchorage-independent assays (Fig. 6I), compared to control cells. To demonstrate that the effects of MAML1 are mediated by the oncogenic activity of Notch1 and Gli1, we performed Notch1 and/or Gli1 silencing in MDA-MB-453-V5-MAML1, which showed a reduced proliferation rate (Supplementary Fig. 4A, B) and long-term survival (Fig. 6J), suggesting that the silencing of Notch1 and Gli1 can reverse MAML1-induced tumorigenicity. Then, to investigate whether Itch silencing can mimic the MAML1 overexpression, Itch knockdown was performed in MDA-MB-453-pHAGE cells. Notably, Itch silencing induces an enhancement in Notch1 and Gli1 protein levels, resulting in cell proliferation (Supplementary Fig. 5A, B) and long-term survival rate (Fig. 6K), comparable to MAML1-induced tumorigenic effects.

These data demonstrate that MAML1 overexpression can exert oncogenic function by inhibiting the tumor suppressor activity of Itch on Notch1 and Gli1 target proteins that, in turn, acquire oncogenic activity, promoting TNBC aggressiveness.

### MAML1 over-expression drives TNBC tumor growth and metastatic spread in vivo

**Fig. 7:**
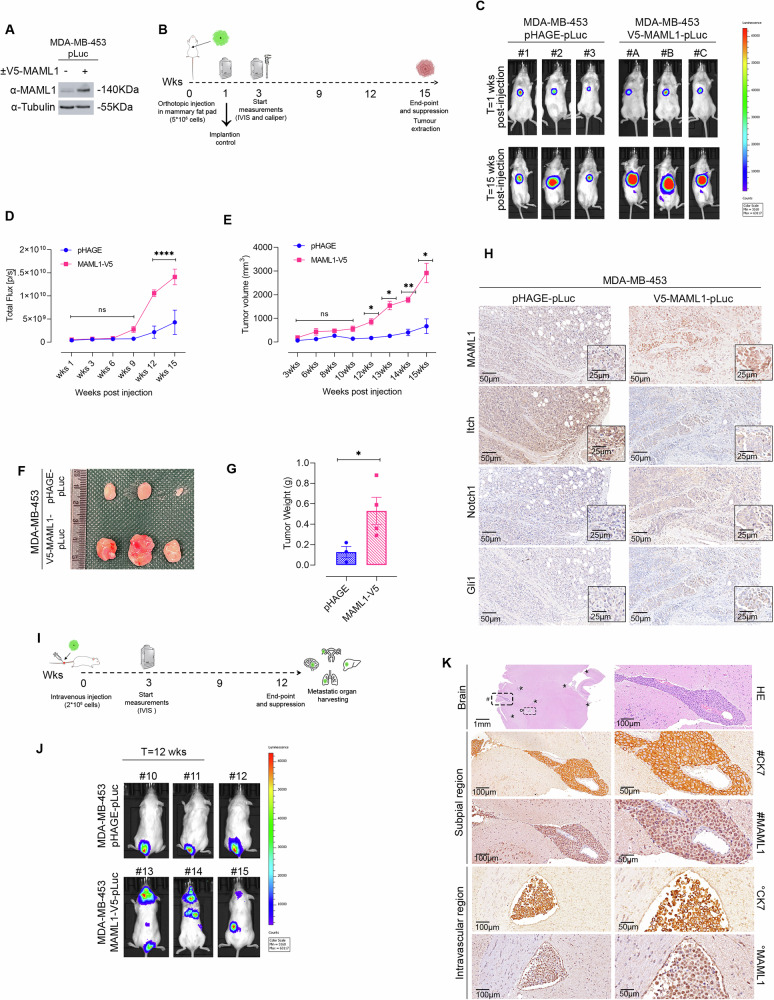
MAML1 empowers tumor growth and multiorgan dissemination in vivo. **A** Immunoblot analysis of MAML1 in MB-453-V5-Maml1 and control cells (MB-453-pHAGE), upon lentiviral infection with vector encoding for pLuc to assess MAML1 expression levels. Tubulin was used as the loading control. **B** Experimental timeline of orthotopic injection of MB-453-V5-MAML1-pLuc and control cells in the mammary fat pad of female NSG mice (5 × 10^6^ cells/each injection) (*n* = 4 mice/group). **C** IVIS imaging of orthotopic implantation described in panel (**B**). The pictures show the images at 1-week post-implantation of tumor cells and the experimental endpoint (15wks). Color scale luminescent signal intensity: blue, the least intense signal; red, the most intense signal. **D** Quantification (total flux, photons per second, p/s) of the bioluminescent signal from the tumor regions in (**C**) is depicted. **E** Analysis of tumor volume. Tumor masses of mice in figure **C** were measured by a caliper along the time curse indicated in the figure. **F** Picture of tumor masses explanted 15 weeks after the implantation. Scale bar: 1 cm. **G** Tumor weight was measured at the experimental endpoint. **H** Representative IHC analysis of MAML1, Itch, Notch1 and Gli1 expression in mice tumor masses from (**C**). Tumors derived from control mice show weak staining compared to the strong positivity for MAML1, Notch1 and Gli1 of MB-453-V5-MAML1-pLuc tumor-derived masses (scale bars: 50 μm and 25 μm). **I** Experimental timeline of intra vein (i. v.) injection of MB-453-V5- MAML1-pLuc and control cells in NSG female mice (2×10^6^ cells/each injection) (*n* = 4 mice/group). **J** IVIS imaging of distant organ metastasis examined at the experimental endpoint, 11 weeks upon i.v. injection. Color scale luminescent signal intensity: blue, the least intense signal; red, the most intense signal. **K** Representative IHC analysis of MAML1in mice metastatic lesions from **J**. Hematoxylin and eosin (HE) reveal brain metastasis composed of epithelial cells (staining for Cytokeratin 7, CK7) with subpial and intravascular localization (scale bars: 1 mm, 100 μm, and 50 μm). Representative IHC picture for Maml1 shows strong positivity of neoplastic cells. Panel **D** and **E** represent Mean ± SEM; n.s. *p* > 0.05; *p < 0.05; **p < 0.01; ****p < 0.0001 calculated by multiple *t* test. Panel **G** shows Mean ± SEM; *p < 0.05; **p < 0.01 calculated by two-tailed unpaired *t* test.

To investigate the oncogenic role of MAML1 in TNBC progression in vivo, we orthotopically implanted MDA-MB-453-V5-MAML1 and control cell lines into mammary fat pads of female NSG mice. Both cell lines were stably transfected with luciferase gene (pLuc) to follow xenograft assays (Fig. 7A). One-week post-inoculation, an in vivo imaging system (IVIS) was performed to visualize the engraftment of tumor cells in mice, following the time-course represented in Fig. 7B. The bioluminescent imaging results showed a significantly accelerated tumor growth in mice implanted with MDA-MB-453-V5-MAML1-pLuc cells, compared to control group (Fig. 7C), as revealed by the quantification of bioluminescent signals (Fig. 7D), and measurements of the tumors volume (Fig. 7E). The tumors sizes derived from MDA-MB-453-V5-MAML1 cells, collected 15 weeks after injection, were at least five times larger than the tumors derived from MDA-MB-453-pHAGE-pLuc cells (Fig. 7F, G). Histological analysis from MDA-MB-453-V5-MAML1-pLuc tumor masses confirmed that high expression levels of MAML1 correlated with a strong decrease in Itch, associated with an increase in Notch1 and Gli1 expression levels, compared to control (Fig. 7H), suggesting that the molecular model proposed here is indeed maintained in vivo conditions. Then, to examine whether the over-expression of MAML1 in the MDA-MB-453-V5-MAML1 cells had greater malignancy potential, we performed tail-vein injection experiments to examine metastatic spread ability. We monitored the spontaneous distant multiorgan metastasis by IVIS imaging following the experimental design represented in Fig. 7I. As shown in Fig. 7J, the metastatic dissemination of MDA-MB-453-V5-MAML1 cells in NSG mice was widespread, and reached all sites commonly affected in human patients, including lungs, ovaries, and brain, faster than control mice. Figure 7K shows representative pictures of metastatic dissemination triggered by MDA-MB-453-V5-MAML1 cells in NSG mice. Histological analysis reveals brain metastasis, composed of epithelial tumor cells, revealed by immunohistochemical staining for cytokeratin 7 (CK7), associated with a strong expression of MAML1. Neoplastic cells were found in the subpial region and within vascular structures, revealing that higher expression levels of MAML1 promote vascular invasion and multiorgan dissemination (Fig. 7K).

Altogether, these data demonstrate that higher expression levels of MAML1 are sufficient to drive faster tumor growth and distant organ metastasis in in vivo experiments, contributing to the accelerated progression and increased metastatic potential of TNBC cells.

## Discussion

The key finding of our study is the identification of a non-canonical role of MAML1 as a post-translational regulator of ubiquitin signaling on Itch target substrates, Notch1 and Gli1, by directly governing Itch self-ubiquitylation. These MAML1-induced events result in strong stabilization of Notch1 and Gli1 that gain oncogenic function and promote TNBC progression.

Itch is an E3 ubiquitin ligase, characterized by the HECT domain and four WW domains, which mediate interaction with PPXY motif-containing target proteins, conferring substrate specificity [27], thus regulating intracellular pathways that control diverse physiological and pathological processes, including cancer [24, 25]. Itch plays distinct roles in cancer, showing oncogenic or tumor suppressor functions [42], depending on the substrate tagged for ubiquitylation, like LATS1/2 in Hippo pathway [43], Gli1 in Hh pathway [18, 19] and Notch1 in its pathway [20, 23]. Itch may be activated by phosphorylation events [44, 45] and by association with adaptors, such as Ndfip1 [46, 47], Numb [18–21] and β-arrestin [48]. In contrast, very little is known about the mechanisms that negatively regulate Itch stability and its catalytic activity [49–51].

In this work, we demonstrate that MAML1 can directly interact with Itch WW domains through the PPQY motif located in the C-terminal domain. We also show that this binding is essential to trigger Itch self-ubiquitylation events through K63-linkage, resulting in a drastic reduction of Itch expression in a dose-dependent manner. Significantly, a single point mutation that disrupts the PPQY motif is sufficient to abolish the interaction between MAML1 and Itch, leading to MAML1 loss-of-function in regulating Itch self-ubiquitylation events. Here, we identify MAML1-dependent new molecular insights that configure MAML1 as a negative regulator of Itch expression/catalytic activity. Increasing amounts of MAML1 show the ability to affect the stability of several potentially oncogenic Itch-target proteins, including Notch1 and Gli1.

Consistently, WT MEFs gene-edited for Maml1 and MEF cells derived from Maml1-depleted (*Maml1*^*–/–*^) mouse model show a drastic reduction in endogenous Itch ubiquitylation levels, associated with strong stability of Itch protein, lastly resulting in a decrease in Itch-substrates, Notch1, and Gli1.

Although MAML1 overexpression has been reported to sustain tumorigenesis processes in several tumor types [32–37], including TNBC [16, 17], the underlying molecular mechanisms are poorly characterized. Interestingly, Notch1 [38] and Gli1 [52] are highly expressed in TNBC and dysregulated Notch and Hh pathways are involved in TNBC progression.

Here, we show that combined over-expression of MAML1/Notch1 or MAML1/Gli1 transcripts is strictly correlated with worse clinical outcomes in terms of OS and RFS in TNBC patients. Furthermore, in a small cohort of TNBC patients, we observed higher expression levels of MAML1 in neoplastic cells, suggesting its role in sustaining TNBC progression. To validate our molecular model, we silenced MAML1 in TNBC cell lines that overexpress MAML1 and unveiled an inverse correlation between Itch and MAML1 expression levels, showing that MAML1 can govern Itch self-ubiquitylation events and its catalytic activity on target proteins, Notch1 and Gli1, also in tumor cells. Conversely, MAML1 ectopic expression in TNBC cell models induces a significant increase in Itch self-ubiquitylation events, triggering a strong activation of Notch and Hh pathways, which promote high cellular growth, increased anchorage-independent capacity, and long-term survival. Of note, MAML1-induced tumorigenicity can be reversed by silencing Notch1 and Gli1. Likewise, Itch silencing can mimic the effects of MAML1 overexpression inducing increased expression of Notch1 and Gli1 oncogenes in TNBC cells with low expression levels of MAML1. Accordingly, MAML1 over-expression promotes faster tumor development into the mammary fat pad of the NSG mouse model with multiorgan metastatic spread, including lungs, ovaries, brain, and vascular structures, suggesting that MAML1 empowers the malignant behavior of cancer cells, carrying out an oncogenic effect.

Our findings unveil new molecular insights for MAML1, which plays a dual role in TNBC. Starting from the well-recognized role as a co-transcriptional factor in numerous signaling pathways, here, we report that MAML1 functions as an Itch-negative regulator, ultimately reinforcing Notch and Hedgehog tumor-promoting signaling pathways that support tumor aggressiveness. These findings indicate that the overexpression of MAML1 can act as a potent cancer driver by simultaneously disrupting multiple signaling pathways, working both as a transcriptional co-activator and post-translational regulator of Itch-targeted proteins, underlying TNBC heterogeneity. Our new mechanistic insights based on MAML1 overexpression may open the way toward novel therapeutic strategies for simultaneously switching off several oncogenic pathways.

## Materials and methods

### Cell culture and treatments

NIH3T3, HEK293T, wild type, and *Ptch1*^*–/–*^ MEFs were cultured in Dulbecco’s modified Eagle’s medium containing 10% fetal bovine serum (Heat-Inactivated; Gibco by Thermo Fisher Scientific, Waltham, MA, USA), 1% penicillin and streptomycin (cat. #P0781, Merck-Sigma Aldrich, Darmstadt, Germania), and 1% L-Glutammine (cat. #G7513, Merck-Sigma Aldrich) as described elsewhere [14]. Primary wild-type and *Maml1*^*–/–*^ MEFs were isolated from E13.5 littermates’ embryos and maintained according to established protocol [14]. MCF10a, MDA-MB-436, HCC1395, HCC1937, BT-549, HS578T, MDA-MB-468, MDA-MB-231, MDA-MB-453, and HCC38 were purchased from American Type Culture Collection, ATCC (Manassas, VA, USA). Routine cell line quality controls were performed on cell lines to evaluate growth characteristics and optimal culture conditions and microbial contaminations (Mycoplasma cat. #G238, Abm Inc., Richmond, BC, Canada) and authenticated by DNA profiling (short tandem repeat, STR) by the cell bank prior to shipping. The cells were maintained according to ATCC recommendations. The media were renewal 2–3 times per week. Cells recovered from frozen aliquots were allowed two passages to reach exponential growth phase following recovery before being used. Cells at passages greater than ten were not used. Cells were treated with 30 μg/ml of Cycloheximide solution (cat. #C4859, Merck-Sigma Aldrich) along a time course. The compound was dissolved in sterile DMSO.

### Cell transfection and plasmids

Transient transfection of HEK293T, NIH3T3 and WT MEFs cell lines was performed using Lipofectamine 2000 (Invitrogen by Thermo Fisher Scientific), according to the manufacturer’s instructions. The following plasmids were described elsewhere: GFP-Gli1 [18], pCS2-HA3-Gli1 [19], pFLAG-CMV-2 MAML1 full-length (1-1016) [53], pFLAG-CMV-2 MAML1 (1-302) and pFLAG-CMV-2 MAML1 (303-1016) [14], p6872 pHAGE-N-V5-MAML1-FL was a gift from Peter Howley (Addgene, MA, USA plasmid # 37048; http://n2t.net/addgene:37048; RRID: Addgene_37048) [54], HA-Ub-WT, HA-Ub-K48R, HA-Ub-K63R [18], pcDNA-Myc-Itch [19], Flag-Itch-WT, Flag-Itch-C830A [18], Notch1-ΔE [23]. For the generation of pFLAG-CMV-2 MAML1-PPQA we used the QuikChange II XL Site-Directed Mutagenesis Kit (cat. #200522, Agilent, Santa Clara, CA, USA) according to the manufacturer instructions. The following primers were designed with QuickCange Prime Design to modify the PPQY motif (CCACCCCAGTAC) in PPQA (CCACCCCAGGCC), in the pFLAG-CMV-2 MAML1 full-length vector:

5′-gtgtcgggtcttgggcctggggtggtgggc-3′

5′-gcccaccaccccaggcccaagacccgacac-3′.

The obtained colonies were subjected to DNA sequencing to assess the aminoacidic change.

### siRNA silencing and CRISPR/Cas9-mediated gene knock-out

Small interference RNA (siRNA) was performed using ON-TARGET plus SMART pool small interference RNA for murine MAML1 (cat. #L-059179-01-0005), human MAML1 (cat. #L-013417-00-0020), human Notch1 (cat. #L-007771-00), human Gli1 (cat. #L-003896-00), human Itch (cat. #L-007196-00-0005) or scrambled control (cat. #D-001810-10-20) purchased by Dharmacon Inc. (Lafayette, CO, USA). The cells were transfected with the above-mentioned siRNA using Lipofectamine RNAiMAX (Invitrogen by Thermo Fischer Scientific), according to the manufacturer’s instructions, for 48 h or 96 h.

MAML1 Knock-out was performed with the (p01)-U6-gRNA:CMV-Cas9-2a-tGFP plasmid targeting MAML1 with two different small guide RNA (sgRNA): GCACAGTTCGATGCGCCGG (sgRNA#1; MISSION™ gRNA ID: MMPD0000107451) and GACCTGCCGTGCATGATCG (sgRNA#2; MISSION™ gRNA ID: MMPD0000107452), compared to Negative control#1, obtained from Merck-Sigma Aldrich and transfected according to the “All-in-one Cas9-reporter Vectors for High Efficiency Single Cell Cloning” protocol provided by the manufacturer. Briefly, after 48 h of transfection, transfected cells were sorted for the Green Fluorescent Protein (GFP) marker by single cell sorting with FACSAria III (BD Biosciences, Franklin Lakes, NJ, USA). Single clones were then seeded in 96-well plates and analyzed by Western Blotting assay.

### Lentiviral infection

For the generation of cell lines stably overexpressing MAML1, pHAGE-N-V5-MAML1-FL or pHAGE_puro vectors were used. pHAGE_puro was a gift from Christopher Vakoc (Addgene plasmid # 11869; http://n2t.net/addgene:118692; RRID: Addgene 118692) [55]. Briefly, HEK293T were transfected with lentiviral vector and packaging plasmids by Lipofectamine 2000 (Invitrogen by Thermo Fisher Scientific), according to manufacturer’s instructions. After 48 h of transfection, the supernatants containing viral particles were collected and used for transduction assays, by using 2 μg/ml of Polybrene (cat. #TR1003-G, Merck-Sigma-Aldrich) [56]. Conditioned medium was maintained for two days at 37°C and then it was replaced by fresh medium. Stable clones were obtained by using 1.5 μg/ml of Puromycin (cat. #P8833, Merck-Sigma-Aldrich) for one week.

Lentiviral construct encoding luciferase pLenti CMV V5-LUC Blast (w567-1) was a gift from Eric Campeau (Addgene plasmid # 21474; http://n2t.net/addgene:21474; RRID: Addgene_21474) [57]. MDA-MB-453 stably expressing MAML1 and control cells were transduced with the viral supernatants produced by transient transfection of lentiviral vector and packaging plasmids into HEK293T. Stable clones were obtained by using 2 μg/ml of Blasticidin (cat. #15205, Merck-Sigma-Aldrich) for one week.

### RT-PCR/qRT-PCR

Total RNA extraction, RT-PCR and qRT-PCR were already described elsewhere [58]. Briefly, upon total RNA extraction with TRIzol Reagent (cat. #15596-018, Invitrogen by Thermo Fisher Scientific), 1 μg of RNA was processed for RT-PCR using SensiFAST™ cDNA Synthesis Kit (Bioline, London, UK). Gene expression analysis was performed by quantitative real-time PCR (qRT-PCR) using Taq-Man designed assays on demand (AoD) (Applied Biosystems by Thermo Fisher Scientific) for the specific target genes on the StepOnePlus™ Real-Time PCR System (Applied Biosystems by Thermo Fisher Scientific), following the manufacturer’s protocol for the comparative C_T_ method. The reaction mix containing the cDNA template (cat. #BIO-65064, Bioline) and the Taqman gene expression assays (Applied Biosystems by Thermo Fisher Scientific) was amplified using standard qPCR thermal cycler parameters. Each sample was amplified in triplicate and the average of the three threshold cycles was used to calculate the number of transcripts. Data were normalized with hypoxanthine guanine phosphoribosyl transferase (*Hprt*) as internal control gene. mRNA quantification was expressed, in arbitrary units, as ratio of sample quantity to the mean value of control sample. The following AoDs were used: Hprt (Mm00446968_m1; Mm01545399_m1); Gli1 (Mm00494654_m1); Ptch1 (Mm00436026_m1); Igf2 (Mm00439564_m1); Hes1 (Mm01342805_m1) and Hey2 (Mm00469280_m1).

### Protein extract and western blot assay

The whole cell extracts (WCEs) were previously described [59]. Briefly, upon washing in phosphate buffer cold (1X PBS) cells were resuspended in a cold lysis buffer containing: 50 mM Tris ‑ HCl pH7.5, 150 mM NaCl, 1% NP-40, 5 mM EDTA pH8, 100 mM NaF, 1 mM PMSF, 1 mM Na3VO4 and 1X protease inhibitor, incubated on ice for 30’ and then centrifuged at 12000 rpm for 30’ at a temperature of 4 °C. The WCE was subsequently quantified according to the Bradford method (Bio-Rad, Hercules, CA, USA). The protein extracts were denatured at 95 °C for 5’ and separated on an acrylamide gel, at different percentages, under denaturing conditions (SDS-PAGE) and, finally, transferred to a nitrocellulose filter (Bio-Rad). The filter was incubated with primary antibodies: α-Flag (cat. #F3165), α-Myc (cat. #M4439), α-β-Actin (cat. #A5441), and α-Tubulin (cat. #T9026) from Merck-Sigma Aldrich; α-HA (cat. #sc-7392), α-GST (cat. #sc-138), α-GFP (cat. #sc-8334), α-Gli1 (cat. #sc-515751), α-Ubiquitin (cat. #sc-8017), α-Vimentin (cat. #sc-373717) and α-N-cadherin (cat. #sc-271386) from Santa Cruz Biotechnology (Santa Cruz, CA, USA); α-Notch1 (cat. #3608), α-Notch1-Val1744 (cat. #4147), α-Gli1 (cat. #2643), α-MAML1 (cat. #11959), α-Itch (cat. #12117), α-V5 (cat. #80076), α-PCNA (cat. #2586); and α-V5 (cat. #13202) by Cell Signaling Technology (Beverly, MA, USA); α-Itch (cat. #611199) by BD Biosciences. Bound antibodies were detected with Western Bright ECL enhanced chemiluminescence (cat. #K-12045-D50, Advansta, San Jose, CA, USA). Uncropped Western blots are provided in Supplementary Material.

### Immunoprecipitation and immunoblot analysis

For co-immunoprecipitation analysis, cells were lysed as above-mentioned, and 500 µg up to 1 mg of WCE was incubated with Protein G-Agarose (cat. #sc-2002) or Protein A-Agarose (cat. #sc-2001) beads by Santa Cruz Biotechnology, for 2 h at 4°. Subsequently, the pre-cleared WCE was incubated 2 h or overnight at 4 °C with specific primary antibodies or IgG used as a control (mouse: cat. #sc-2025, Santa Cruz Biotechnology; rabbit: cat. #12-370, Merck-Millipore, Darmstadt, Germania). Then, the formed immunocomplexes were incubated with G- or A-Protein agarose beads for 1 h at 4 °C, washed extensively with wash buffer, and samples were prepared for SDS-PAGE resolving and then the interaction was evaluated by Western blot analysis, as described above. Co-immunoprecipitation with α-Flag M2 affinity gel agarose (cat. #A2220) or α-HA-Agarose (cat. #A2095) was performed with Mouse IgG-Agarose (cat. #A0919) as control. They were provided by Merck-Sigma-Aldrich. Uncropped Western blots are provided in Supplementary Material.

### In vitro ubiquitination assay

Where indicated the cells were transfected with the indicated plasmids. TNBC cell lines silenced for MAML1 were transfected with HA-Ub vector, upon 24 h of MAML1 silencing. Analyzed cells were lysed with RIPA buffer (50 mM Tris-HCl at pH 7.6, 150 mM NaCl, 0.5% Sodium Deoxycholate, 5 mM EDTA, 0.1% SDS, 100 mM NaF, 2 mM Sodium Pyrophosphate, 1% NP-40) supplemented with protease and phosphatase inhibitors or denaturing buffer (2% SDS, 50 mM Tris at pH 7.5, 0.5 mM EDTA, 1 mM DTT) to disrupt protein/protein interactions and diluted 10 times with lysis buffer and subjected to immunoprecipitation with antibodies indicated in figures for 2 h or overnight. The immunoprecipitated proteins were then washed five times with the washing buffer, resuspended in sample loading buffer, boiled for 5 min, resolved in SDS-PAGE, and then subjected to immunoblot analysis. Ubiquitinated forms were detected by immunoblot analysis. Where indicated, wild-type ubiquitin was substituted with ubiquitin mutants (K48R and K63R). Uncropped Western blots are provided in Supplementary Material.

### GST pulldown assay

The following recombinant GST-fusion proteins were expressed in Escherichia coli BL21 and purified as already described [60]: GST, GST-Itch, GST-4WWs and GST-HECT. WCEs of transfected HEK293T were subjected to a preclearing protocol using glutathione beads (cat. #10-0756-01, GE Healthcare, Chicago, II, USA). Recombinant proteins were bound to the Glutathione Sepharose 4B beads and incubated for 2 h with the precleared whole cell extracts. Samples were washed with GST lysis buffer (20 mM TRIS HCL-ph8, 200 mM NaCl, 1 mM EDTA, 0.5X NP-40, 25 µM PMSF, 1 mM Na3VO4, 1X Protease Inhibitor) four times and the immunocomplexes were eluted from the Glutathione Sepharose 4B beads through the addition of reduced Glutathione pH 8. Finally, samples were separated through SDS-PAGE and analyzed by immunoblotting. Uncropped Western blots are provided in Supplementary Material.

### Proximity ligation assay (PLA)

In situ PLA with the Duolink® In situ-Fluorescence Technology, Olink® Bioscience (Merck-Sigma-Aldrich) was already described elsewhere [61]. Briefly, MDA-MB-453 stably expressing V5-MAML1 and control cells, MDA-MB-231 and MDA-MB-436 (upon 48 h of MAML1 silencing) were used. All the steps were performed according to the manufacturer’s protocol. The primary antibodies used were α-Itch (cat. #PA5-116452, Invitrogen by Thermo Scientific) and α-Ubiquitin (cat. #ST1200, Merck-Millipore). Hybridization between the two PLA α-rabbit PLUS and α-mouse MINUS probes leading the fluorescent red signal only occurs when the distance between the two antigens is less than 40 nm. Single plane confocal images were acquired using a Zeiss LSM 980 confocal microscope with a 63×/1.35NA oil-immersion objective (Zeiss). Analysis of PLA intensity was executed with Zeiss Zen System Software.

### Immunofluorescence and confocal imaging

Immunofluorescence staining of MDA-MB-453 stably expressing V5-MAML1 and control cells was performed as described elsewhere [62, 63]. The cells were stained with α-Itch (cat. #PA5-116452, Invitrogen by Thermo Fisher Scientific) and the secondary antibody Alexa Fluor 488-conjugated goat-α-rabbit (cat. #A11008, Invitrogen by Thermo Fisher Scientific). Nuclei were counterstained with Hoechst reagent (cat. #33342, Invitrogen by Thermo Fisher Scientific). Single plane confocal images of the cell were acquired using a Zeiss LSM 980 confocal microscope with a 63×/1.35NA oil-immersion objective (Zeiss, Oberkochen, Germany).

### Cell viability assay

As already described [64], MDA-MB-453 stably expressing V5-MAML1 and control cells were seeded in 12-well plate at 1*10^5^ cells; 5*10^5^ MDA-MB-436 and MDA-MB-231 cells were plated onto a 6-well plate and small interference RNA (siRNA) was performed using ON-TARGET plus SMART pool small interference RNA for human MAML1 or scrambled control, as described above. Trypan blue (cat. #T8154, Merck-Sigma-Aldrich) cell counting was used to determine the number of viable cells, at different time points or after 96 h from transfection. Growth of cells was calculated relative to control cells. Measurements were performed in technical triplicates and figures show the averages ± SEM of at least 3 biological replicates. Single plane images were acquired using a Zeiss LSM 980 confocal microscope with a 40×/0.6 Korr Ph2 objective (Zeiss). Analysis of PLA intensity was executed with Zeiss Zen System Software. The fraction of Area occupied by cells was calculated from brightfield microscopy images at different times point, using the “Analyze particles” tool in Fiji-ImageJ Software. Images were thresholded and binarized. Particles were analyzed with defined size (50-∞ pixels^2^) and circularity (0.30-1.00) parameters to exclude artifacts. The fraction of area covered by cells was calculated as the ratio between the total area of detected particles and the total image area and expressed as a percentage.

### MTS assay

To analyze the cell growth rate an opportune amount of cells per well were plated onto a 96-well plate. MDA-MB-436 and MDA-MB-231 cells were plated onto a 96-well plate and small interference RNA (siRNA) was performed using ON-TARGET plus SMART pool small interference RNA for human MAML1 or scrambled control, as described above. MDA-MB-453 cells overexpressing V5-MAML1 and the control cells were analyzed 48 h after plating. The CellTiter 96(R) AQueous One Solution Assay (cat. #G3580, Promega, Madison, WI, USA) was added to each well according to the manufacturer’s instructions. Spectrophotometric absorbance at 490 nm wavelength was determined by the plate reader GloMax-Multi Detection System (Promega).

### Colony formation assay

MDA-MB-453 stably expressing V5-MAML1 and control cells were seeded in the appropriate density in 24-well plates. 15 days after seeding, the colonies were fixed with a solution of 90% methanol and 10% acetic acid, at room temperature for 10’ [64]. Then, they were visualized by a staining solution of 0.1% crystal violet (cat. #27335.01, SERVA, Heidelberg, Germany) diluted in methanol for 3’. After staining, plates were washed with water and left to dry overnight. Finally, plates were scanned and stored. Analysis of the Area Percent and Intensity Percent of colonies was performed with Fiji-ImageJ software and the statistical analysis with GraphPad Prism.

### Soft agar assays

Soft agar assay was performed as already described elsewhere [65]. In a 6-well plate pre-coated with 0.6% soft agar (cat. #A9414, Merck-Sigma-Aldrich) dissolved in complete medium. MDA-MB-453 stably expressing V5-MAML1 and control cells were plated on the upper layer (0.4% agarose dissolved in cell medium) and covered by 1 ml of complete medium. Upon colony formation, the latter were fixed with 10% Methanol/10% Acetic Acid for 10’ and then stained with a 0.005% Crystal Violet (cat. #27335.01, SERVA).

### Animal studies

The generation and typing of *Maml1*^*–/–*^ mouse have been described elsewhere [14, 31]. Mice were already available in our animal husbandry and maintained on a C57BL/6 background. The six-week-old female NSG (NOD.Cg-*Prkdc*^*scid*^ *Il2rg*^*tm1Wjl*^/SzJ) mice were purchased from Envigo RMS Italia. Mice were housed in the Institute’s Animal Care Facilities in accordance with Italian Governing Law (D.Lgs. n.26/2014/ Protocol Number: C1368.17 and C1368.22) and European Directive 2010/63/UE.

For in vivo orthotopic models (*n* = 4 mice/group), NSG mice were anesthetized by intraperitoneal (i.p.) injection of ketamine (10 mg/kg) and xylazine (100 mg/kg). Subsequently, a suspension of MDA-MB-453 V5-MAML1 and control cells (MDA-MB-453 pHAGE), infected with the lentiviral construct encoding luciferase pLENTI-CMV-Blast-LUC (pLuc) as described above, were prepared. Briefly, cells were resuspended in 200 µl of PBS at a density of 5*10^6^/200 µl. Injection of cells occurred into the mammary gland 3 fat pad with a 30 G, 1/2” needle [66].

For in vivo study of metastasis (*n* = 4 mice/group), we performed an intravenous (i.v.) tail injection on NSG mice. Upon vasodilatation of the veins of mice by a heat lamp, the animals were placed in a restrainer device and injected with a suspension of MDA-MB-453 V5-MAML1 or control cells (MDA-MB-453 pHAGE), infected with pLuc lentiviral construct. Specifically, cells were resuspended in 200 µl of PBS at a density of 2*10^6^/200 µl and injected through a 30 G, 1/2” needle [67].

Bioluminescence imaging was performed upon intraperitoneal injection of 150 mg/kg IVISbrite D-Luciferin Bioluminescent Substrate in RediJect Solution (XenoLight) (cat. ##770504, Revvity Health Sciences, Waltham, MA, USA) using a 25 G needle. After 10 minutes, mice were anesthetized and placed in the IVIS Lumina III In Vivo Imaging System (Caliper Life Science, Hopkinton, MA, USA) chamber for imaging. All mice were scanned under exposure conditions of 30 s (orthotopic models) and 120 s (i.v. injections), along a time course, as indicated in the figures. Data acquisition and analysis were performed using the Living Image software (Caliper Life Science). Results are expressed as the total flux (photons/sec) in the ROI of mice tumors.

The in vivo experiments were terminated at the human endpoint and at sacrifice, the tumor masses and mice organs were collected and processed for further analysis.

### Immunohistochemistry (IHC)

For IHC analysis, tissue derived from animal studied and TNBC patients (already obtained for diagnostic purposes) were collected and fixed in 4% formalin and paraffin embedded, as already described [14, 65]. Consecutive sections (2 μm thick) were stained with Hematoxylin and Eosin (H&E) and the immunocytochemical assay was performed with α-MAML1 (dilution 1:50; cat. #PA5-111083), α-Itch (dilution 1:500; cat. #PA5-116452) and α-human CK7 (dilution 1:100; cat. #MA1-06316) antibodies from Invitrogen by Thermo Fisher Scientific; α-Notch1 (dilution 1:200; cat. #3608 by Cell Signaling Technology) and α-Gli1 (dilution 1:150; cat. #sc-515751 by Santa Cruz Biotechnology). Detection was carried out with UltraTek HRP (cat. #AFK600, ScyTek Laboratories, Logan, UT, USA) and DAB as chromogen (Impact DAB EqV Substrate Kit, Vector Laboratories, Newark, CA, USA), according to manufacturer’s instructions. Images were acquired with Ventana DP200 slide scanner (Roche, Basil, Switzerland). Any degree of cytoplasmic and/or nuclear staining was considered positive. For each case, tumor staining was semi-quantitatively assessed using the H-score method, as follows: H-score = (3 x percentage of tumor cells with 3+ staining) + (2 x percentage of tumor cells with 2+ staining) + (1 x percentage of tumor cells with 1+ staining). This score, therefore, is in the range of 0 to 300. H-scores for MAML1 immunohistochemical staining were also categorized into three expression levels: low (0 to 100), intermediate (>100 to 200), or high (>200 to 300).

### Datasets and in silico analyses

Clinical information and gene expression levels for Breast Cancer patients were derived from the Molecular Taxonomy of Breast Cancer International Consortium (METABRIC) [68–70] and downloaded from cBioportal site (https://www.cbioportal.org/). Breast Invasive Ductal Carcinoma patients with Triple Negative Breast Cancer (TNBC) and who had a minimum follow-up of 1 year were selected and included in the analysis (*n *= 261). For the analysis of both Overall Survival (OS) and Relapse-Free Survival (RFS), the patients were stratified into two groups on the basis of the MAML1/NOTCH1 and MAML1/GLI1 signature expression or the single gene. The gene signature score was calculated as the average of the Z-scores for MAML1 and NOTCH1 mRNA expression in the MAML1/NOTCH1 signature and for MAML1 and GLI1 mRNA expression in the MAML1/GLI1 signature. Then, the patients were stratified into two groups on the basis of the signature score, using the higher tertile as threshold. Survival curves (OS and RFS) were estimated using the Kaplan–Meier method, and statistical differences were tested using the logrank test; *p* value < 0.05 was considered to be statistically significant.

Clinical information and gene expression data for 137 invasive ductal carcinoma TNBC patients who had a minimum follow-up of 1 year from The Cancer Genome Atlas (TCGA) [71] were obtained from the UCSC Toil RNA-Seq Recompute dataset [72] and downloaded using the Xena browser. Patients were stratified into two groups based on the MAML1 gene expression levels, using the higher tertile as threshold. Pearson correlation coefficient (r) was used to assess the linear relationship between MAML1 and Notch1 or Gli1 gene expression levels, in 137 invasive ductal carcinoma TNBC patients from the TCGA dataset. The coefficient (r) ranges from –1 to +1, with values closer to 1 or –1 indicating a stronger relationship. A positive r-value suggests a positive linear relationship, while a negative r-value suggests a negative linear relationship. A value of 0 indicates no linear relationship. Statistical differences were tested using the Wilcoxon test and *p* value < 0.05 was considered to be statistically significant.

### Statistical analysis

All Results were reported as the mean ± S.E.M. (standard error of the mean) or ±S.D. (standard deviation) of at least three independent experiments, each performed in triplicate. Statistical analysis was performed by GraphPad Prism Software. The statistical test employed in each experiment is indicated in the respective figure legends. A *p* value < 0.05 was considered statistically significant (n.s. *p* > 0.05; *p < 0.05; **p < 0.01; ***p < 0.001; ****p < 0.0001).

## Supplementary information

Supplementary Figures

Uncropped Western Blots

## Data Availability

All data in this study are available within the article and Supplementary Information or from the corresponding authors on reasonable request. The datasets analyzed during the current study are available in the Molecular Taxonomy of Breast Cancer International Consortium (METABRIC) [68–70] repository and downloaded from cBioportal site (https://www.cbioportal.org/). The Cancer Genome Atlas (TCGA) datasets [71], used in this study, were obtained from the UCSC Toil RNA-Seq Recompute dataset [72].
