# Supplementary material for: MAML1 drives Notch and Hedgehog oncogenic pathways by inhibiting Itch activity in triple-negative breast cancer
Source: Cell Death Differ. 2025 Nov 21;33(5):971–87. doi: 10.1038/s41418-025-01613-5 (PMC13156293; doi:10.1038/s41418-025-01613-5)
Supplement: Supplementary file 1 — Supplementary Figures [file 41418_2025_1613_MOESM1_ESM.pdf]

Supplementary Figures

MAML1 drives Notch and Hedgehog oncogenic pathways by inhibiting Itch activity in triple-negative breast cancer

Sabrina Zema, Francesca Di Fazio, Maria Pelullo, Sara Di Savino, Bruna Cerbelli, Martina Leopizzi, Laura Di Magno, Carmine Nicoletti, Giovanna Peruzzi, Daniel D'Andrea, Maria V Giuli, Samantha Cialfi, Biagio Palmisano, Alice Turdo, Rocco Palermo, Giulia d'Amati, Gianluca Canettieri, Antongiulio Faggiano, Lucia Di Marcotullio, Matilde Todaro, Isabella Screpanti, Claudio Talora, Saula Checquolo & Diana Bellavia

Supplementary Figure 1. MAML1, Notch1, and Gli1 expression levels are correlated in TNBC patients.

Supplementary Figure 2. Kaplan-Meier curves for TNBC patients based on individual expression levels of MAML1, Notch1, and Gli1.

Supplementary Figure 3. MAML1 overexpression sustains proliferation events in TNBC cells.

Supplementary Figure 4. Silencing of Notch1 and Gli1 reverses the malignant effects of MAML1 overexpression in TNBC cells.

Supplementary Figure 5. Itch knockdown mimics the effects of MAML1 overexpression.

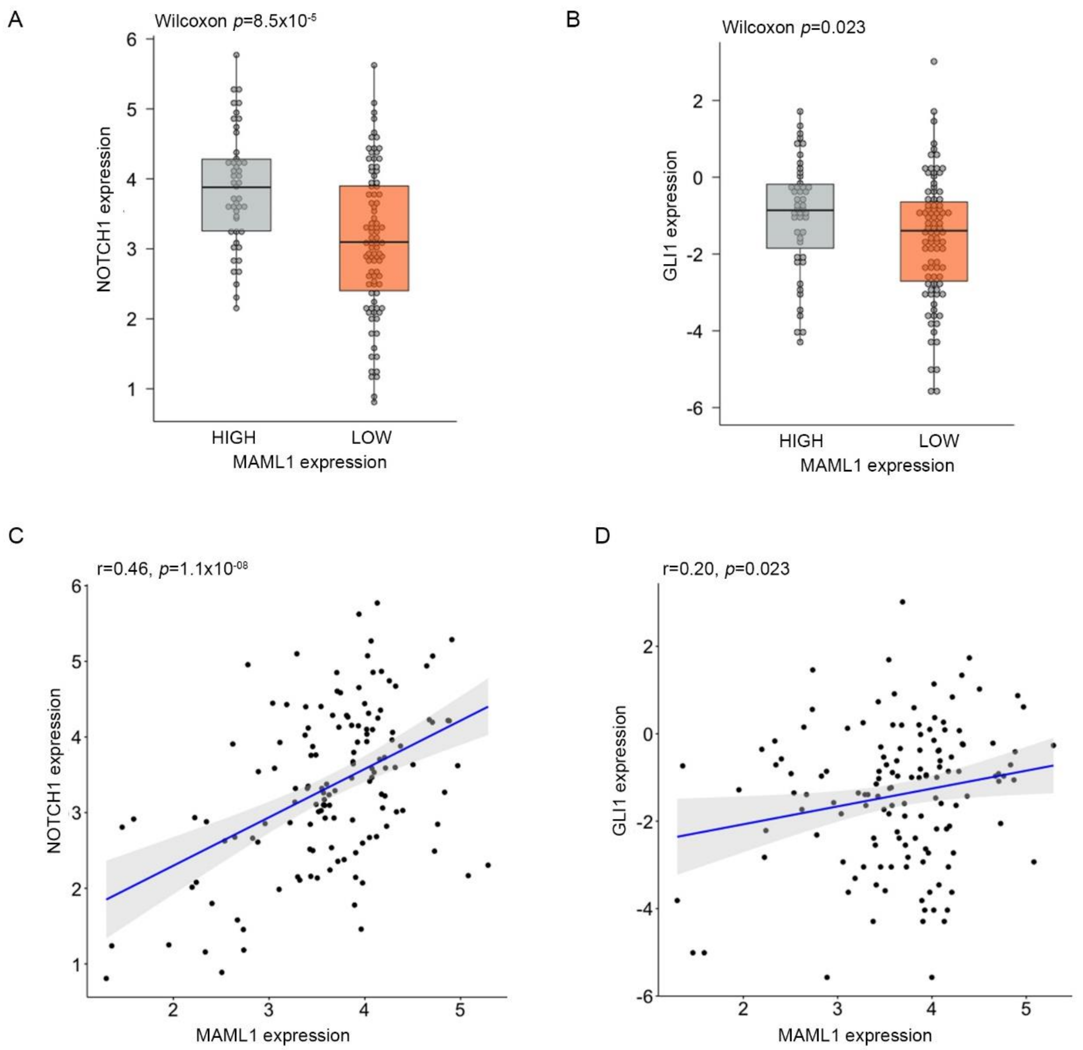

Supplementary Figure 1. MAML1, Notch1, and Gli1 expression levels are correlated in TNBC patients. A-B Box plots showing Notch1(left) and Gli1 (right) expression levels in 137 invasive ductal carcinoma TNBC patients from The Cancer Genome Atlas (TCGA) stratified into two groups (HIGH, n=47 and LOW, n=90) on the base of MAML1 gene expression levels, using the higher tertile as threshold. Expression values are represented as TPM. Statistical differences were tested using the Wilcoxon test and p value < 0.05 was considered to be statistically significant. **C-D** Scatter plots showing the correlations of MAML1 mRNA expression with both Notch1 (left) and Gli1 (right) mRNA expression in 137 invasive ductal carcinoma TNBC patients from the TCGA dataset. Expression values are represented as TPM. Pearson's correlation values and p values are shown.

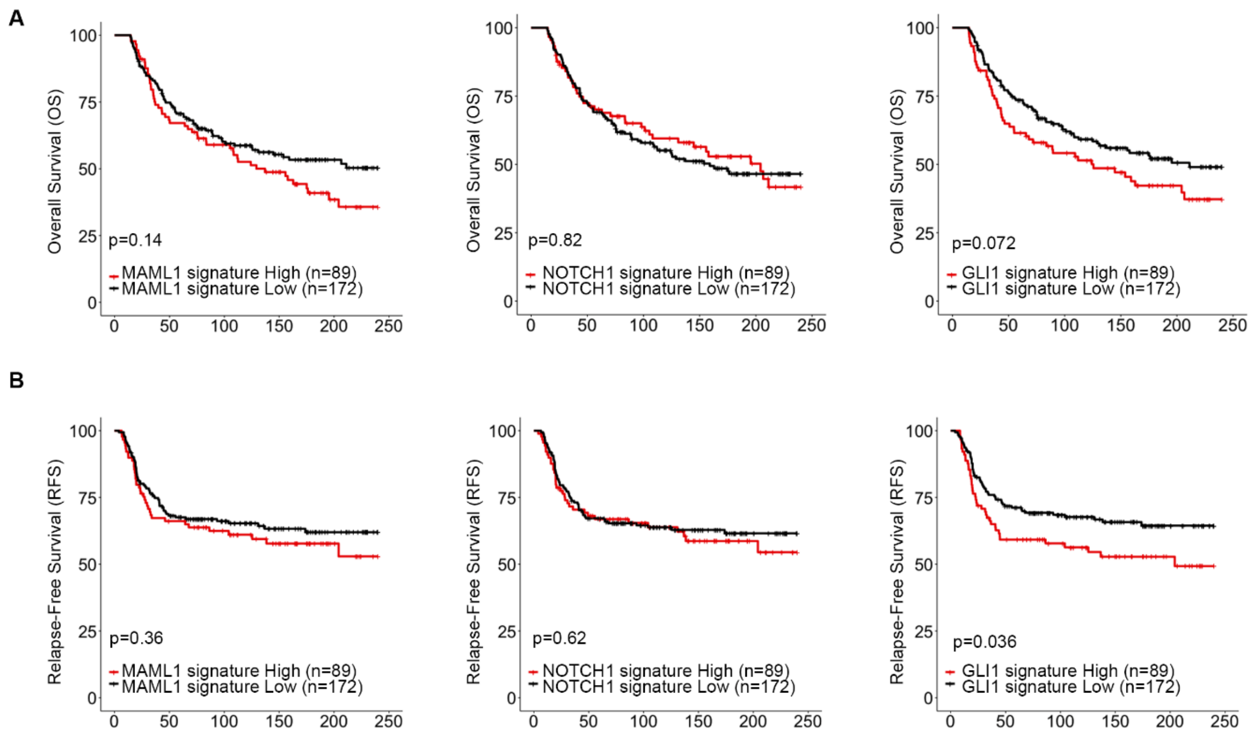

Supplementary Figure 2. Kaplan-Meier curves for TNBC patients based on individual expression levels of MAML1, Notch1, and Gli1. A-B Overall Survival (OS) (**A**) and Relapse-Free Survival (RFS) (**B**) Kaplan-Meier curves in Triple Negative Breast Invasive Ductal Carcinoma patients (n =261) from METABRIC database. Patients were stratified in two groups, High and Low MAML1, Notch1 and Gli1 signature, based on the expression levels of the analyzed gene (left panel: MAML1; central panel: Notch1; right panel:Gli1), using the higher tertile as threshold. Statistical significance was assessed using the log-rank test. The *p* values and the number of patients in each group are shown in the corresponding panels.

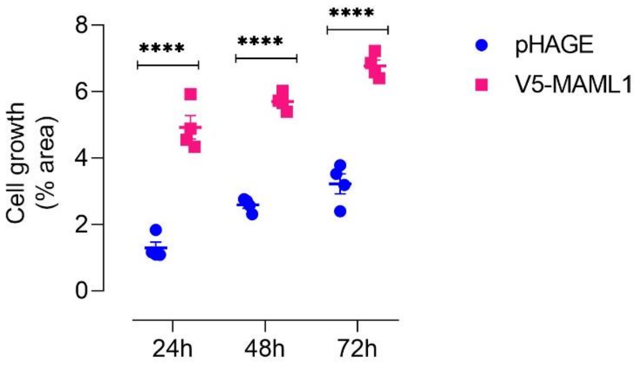

Supplementary Figure 3. MAML1 overexpression sustains proliferation events in TNBC cells. Statistical analysis of pictures presented in Fig. 6E, shown as a graph of cell-covered area fraction. Data represent Mean ± SEM of *n* =4 independent experiments. ****p value *<0.0001* calculated with Multiple t-test.

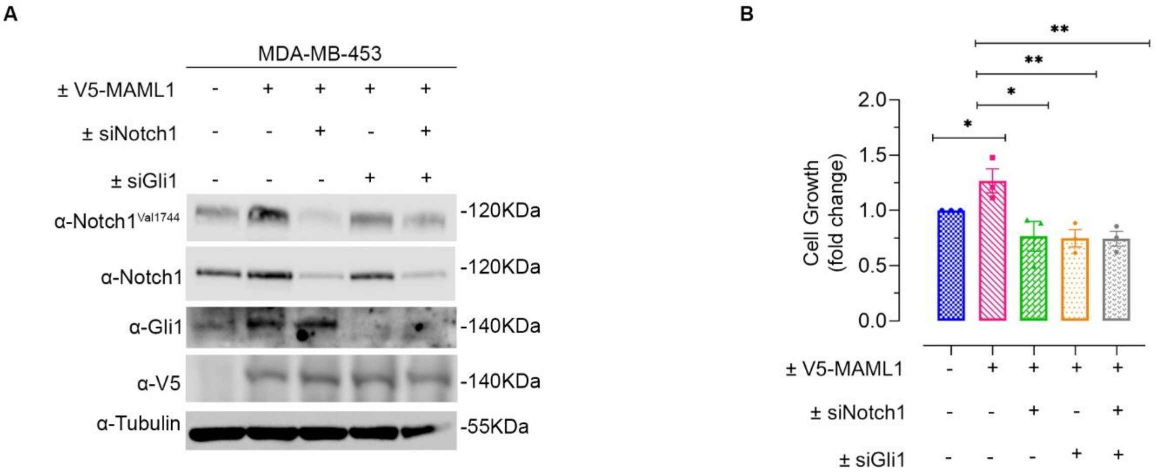

Supplementary Figure 4. Silencing of Notch1 and Gli1 reverses the malignant effects of MAML1 overexpression in TNBC cells. A Representative Immunoblots for Notch1^{Val1744}, Notch1, Gli1 and V5-tagged MAML1 of MDA-MB-453 overexpressing MAML1, upon small interference RNA of Notch1 and/or Gli1 (siNotch1 and/or siGli1), as indicated in the upper panel, compared to control cells (MDA-MB-453-pHAGE siScr; MDA-MB-453-V5-MAML1 siScr). Tubulin was used as a loading control. **B** Analysis of cell count in MDA-MB-453 overexpressing MAML1, upon small interference of Notch1 and/or Gli1, compared to control cells (MDA-MB-453-pHAGE siScr; MDA-MB-453-V5-MAML1 siScr). Data, indicated as fold change, represent Mean ±SEM of $n = 3$ independent experiments. *$p < 0.05$; **$p < 0.01$ calculated with two-tailed unpaired t-test. Representative immunoblotting of $n = 3$ biological replicates with similar results is shown in panel A.

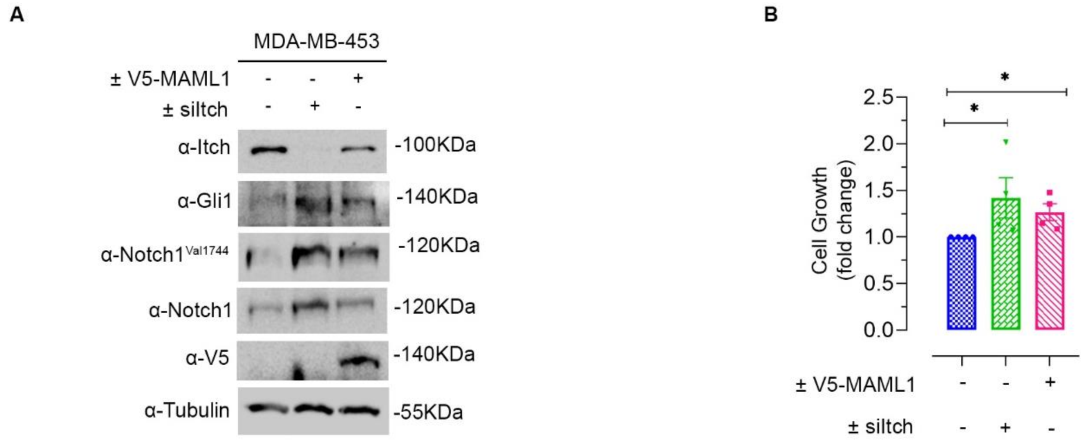

Supplementary Figure 5. **Itch knockdown mimics the effects of MAML1 overexpression. A** Representative Immunoblots for Itch, Gli1, Notch1^{Val1744}, Notch1 and V5-tagged MAML1 of MDA-MB-453-pHAGE cells, upon small interference RNA of Itch (siItch) as indicated in the upper panel, compared to control cells (MDA-MB-453-pHAGE siScr; MDA-MB-453-V5-MAML1 siScr). Tubulin was used as a loading control. **B** Analysis of cell count in MDA-MB-453 pHAGE, upon small interference of Itch, compared to control cells (MDA-MB-453-pHAGE siScr; MDA-MB-453-V5-MAML1 siScr). Data, indicated as fold change, represent Mean ±SEM of n=4 independent experiments. *$p < 0.05$; **$p < 0.01$ calculated with two-tailed unpaired t-test. Representative immunoblotting of n = 3 biological replicates with similar results is shown in panel A.
