# Supplementary material for: MAML1 drives Notch and Hedgehog oncogenic pathways by inhibiting Itch activity in triple-negative breast cancer
Source: Cell Death Differ. 2025 Nov 21;33(5):971–87. doi: 10.1038/s41418-025-01613-5 (PMC13156293; doi:10.1038/s41418-025-01613-5)

Fig 1A

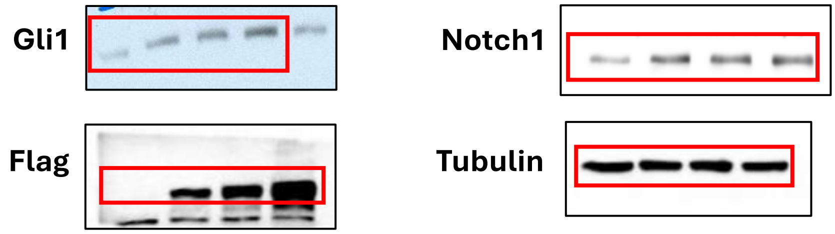

Fig 1B

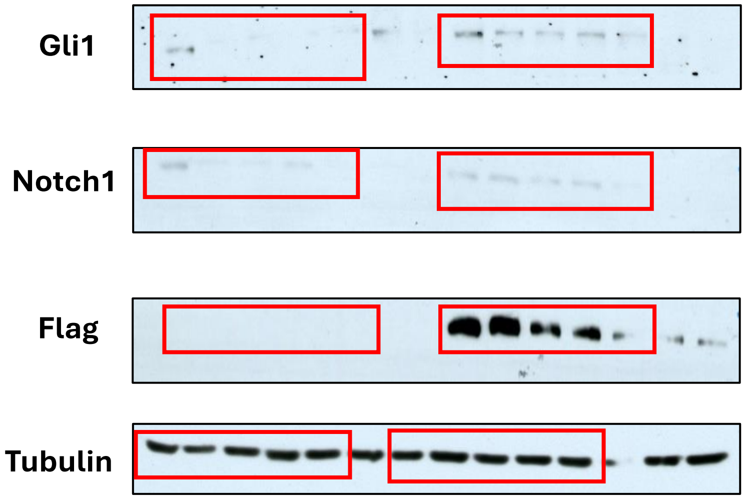

Fig 1D

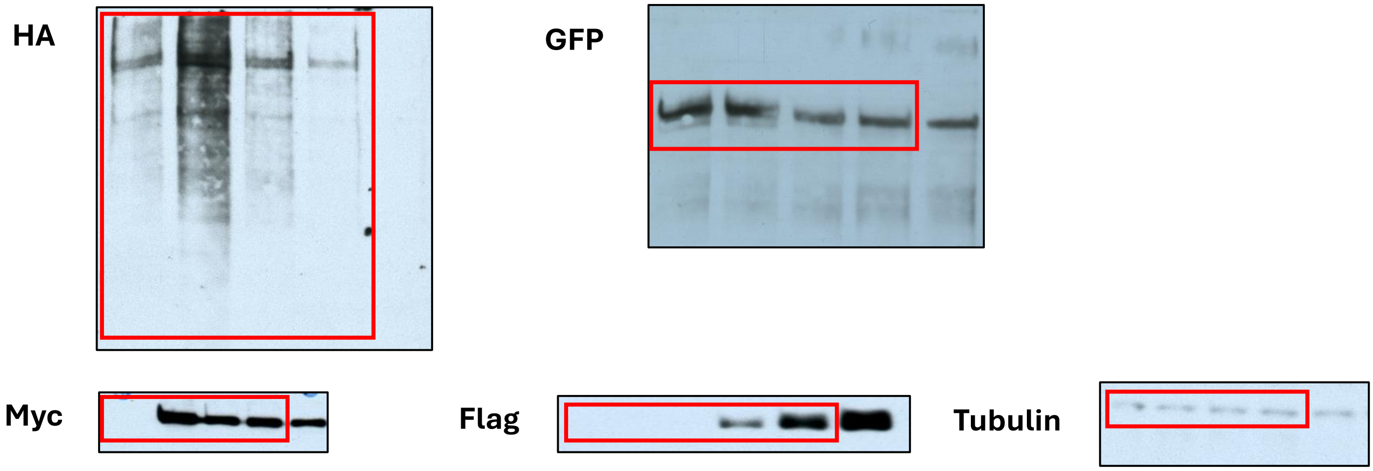

Fig 1E

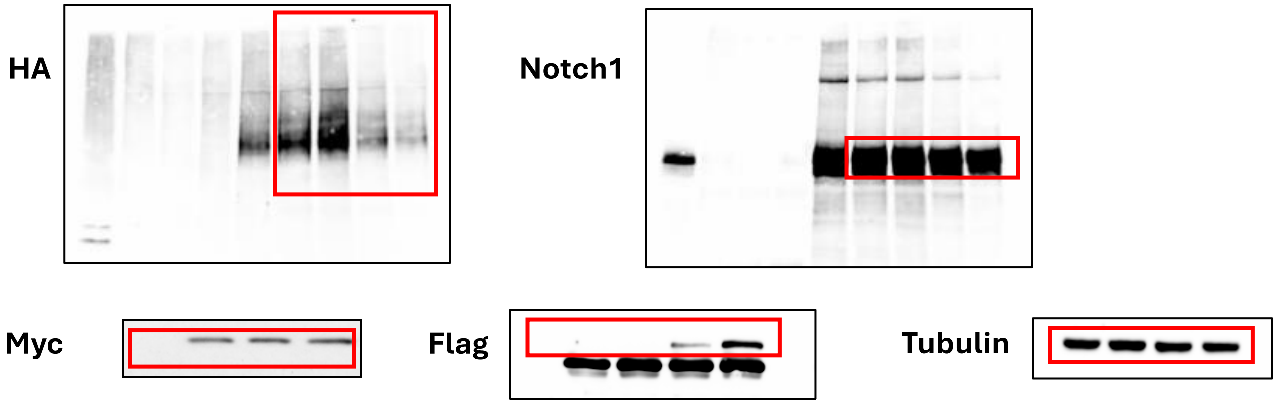

Fig 1F

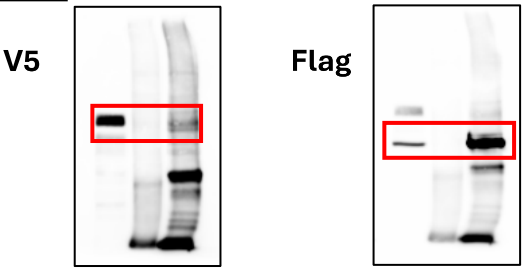

Fig 1G

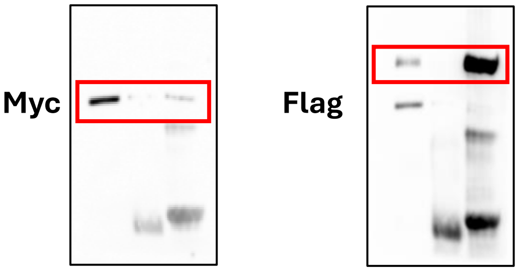

Fig 1H

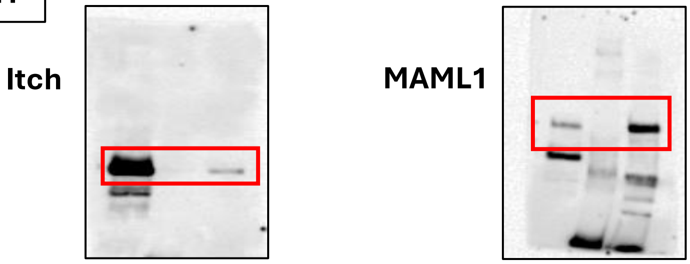

Fig 2A

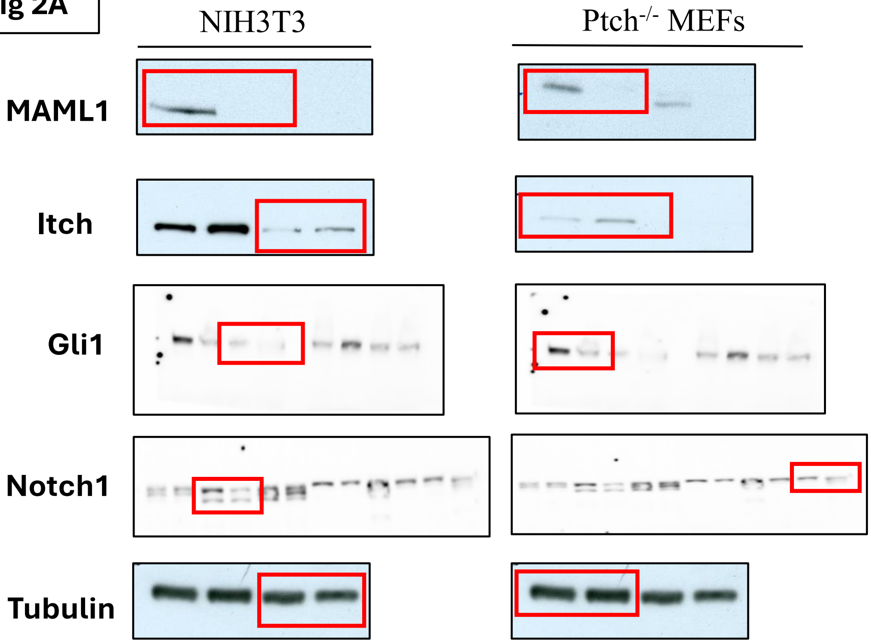

Fig 2B

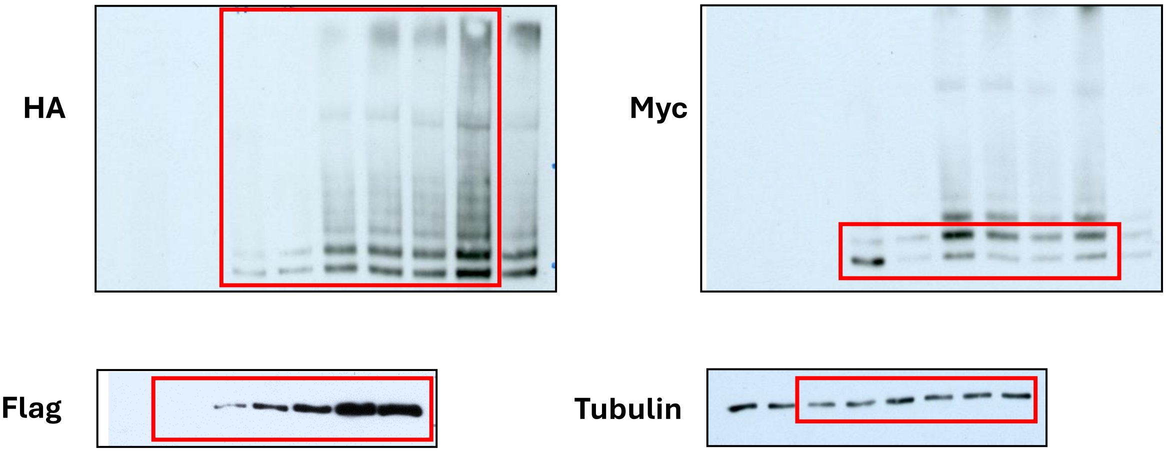

Fig 2C

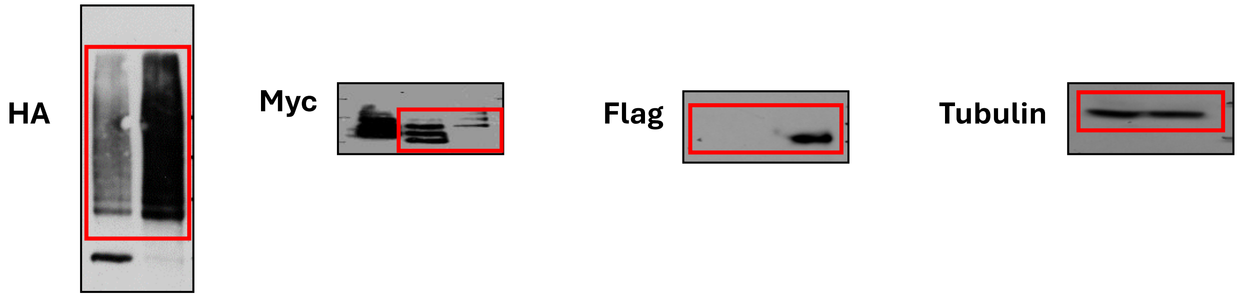

Fig 2D

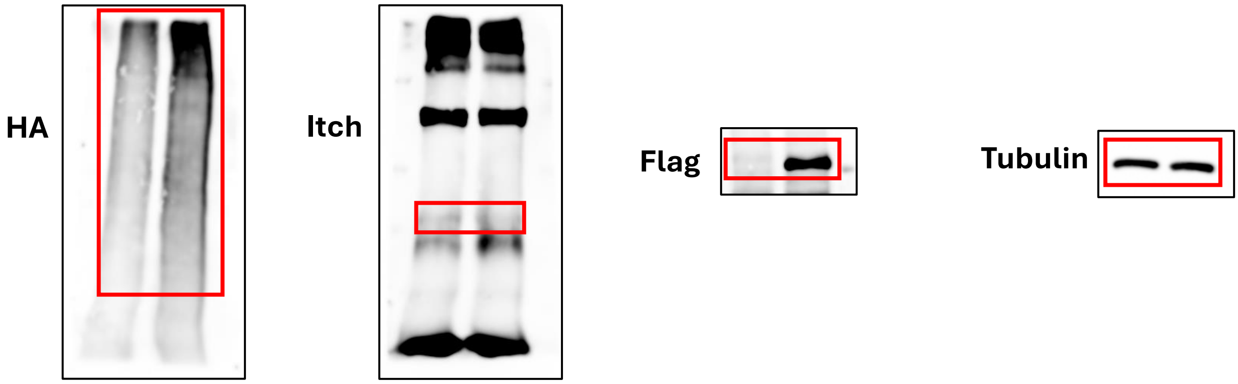

Fig 2E

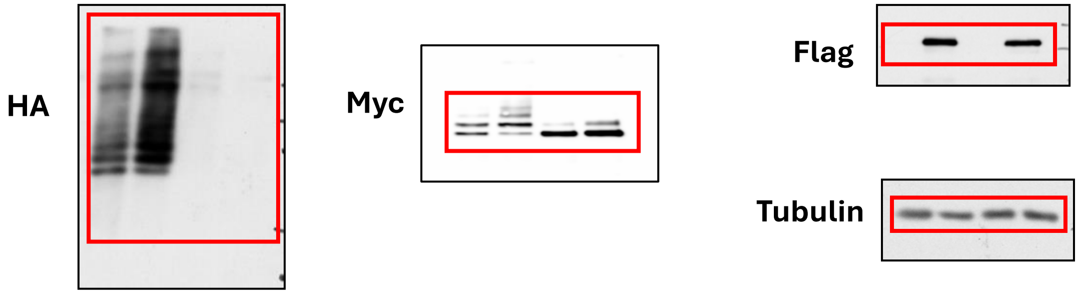

Fig 2F

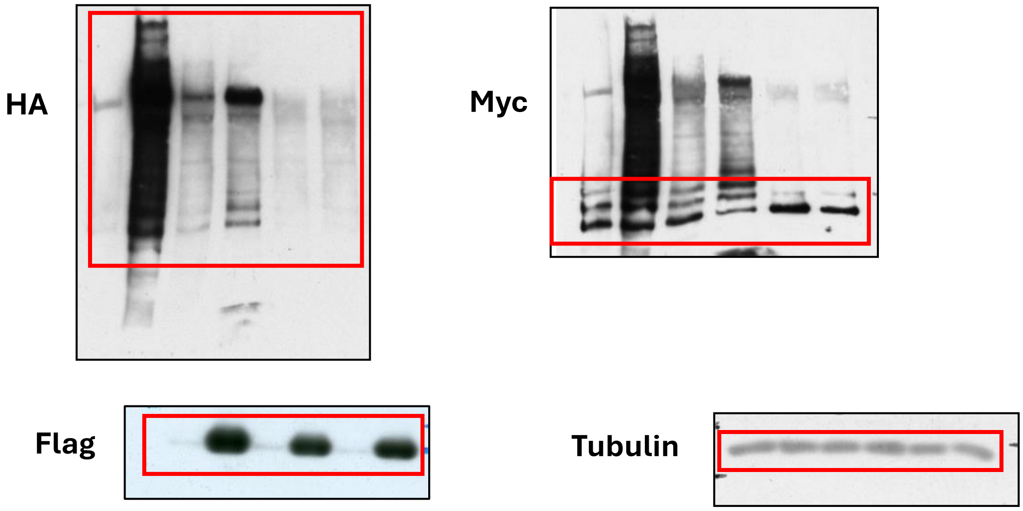

Fig 3A

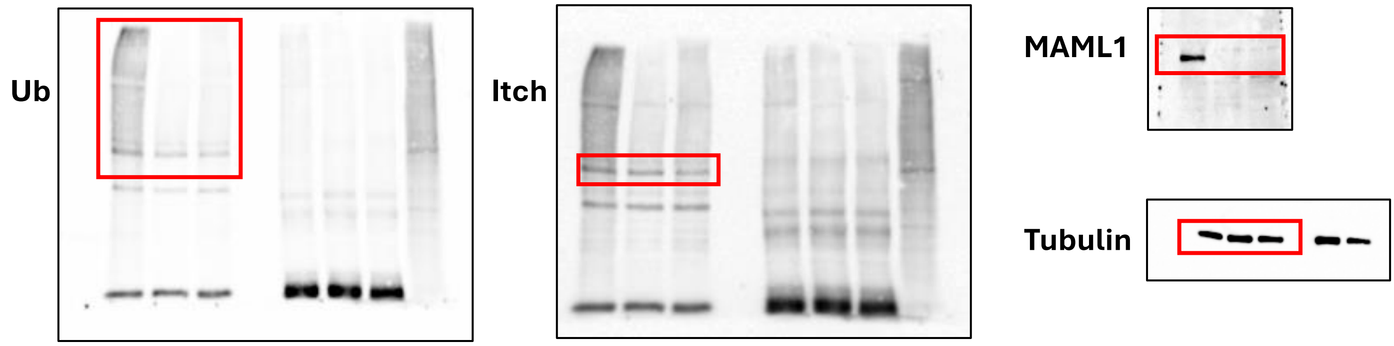

Fig 3B

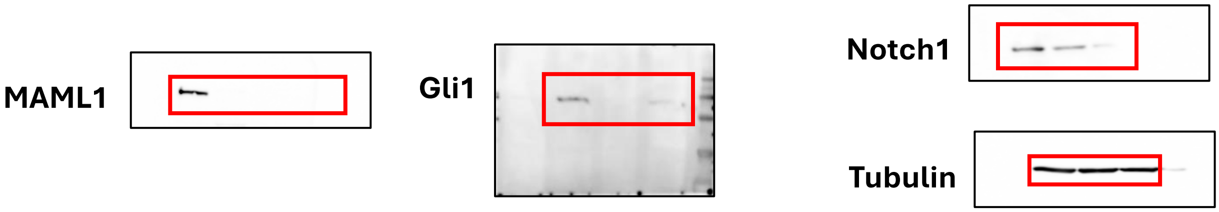

Fig 3D

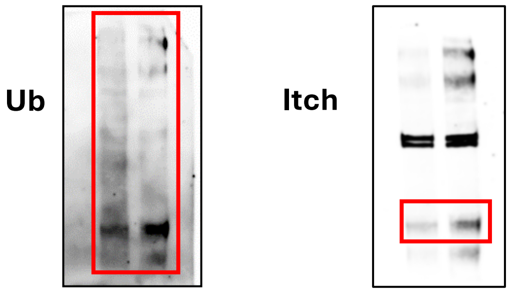

Fig 3E

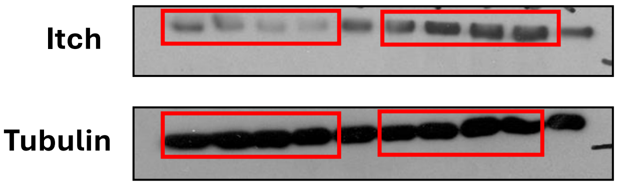

Fig 3F

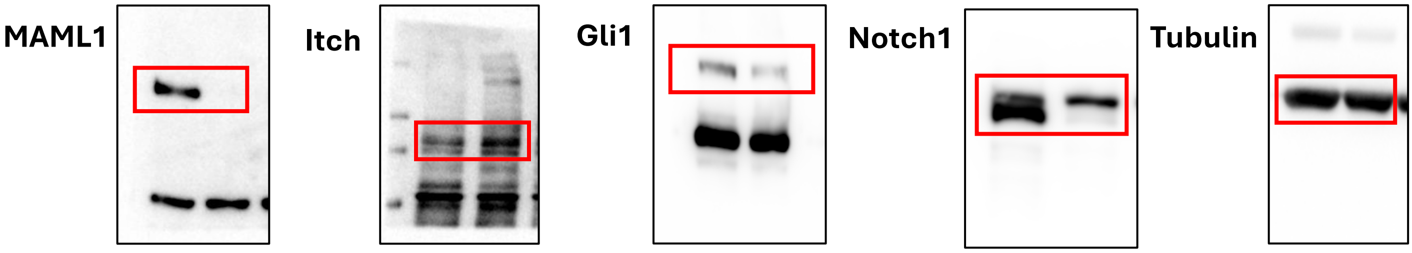

Fig 4B

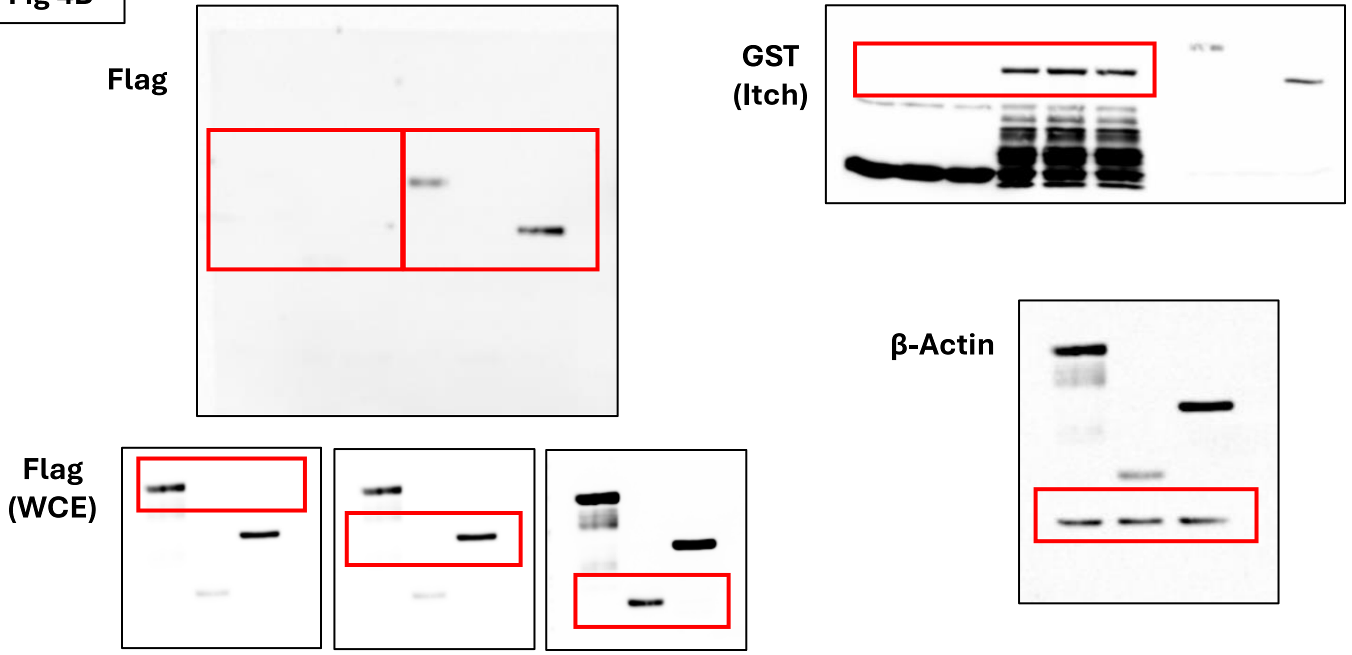

Fig 4C

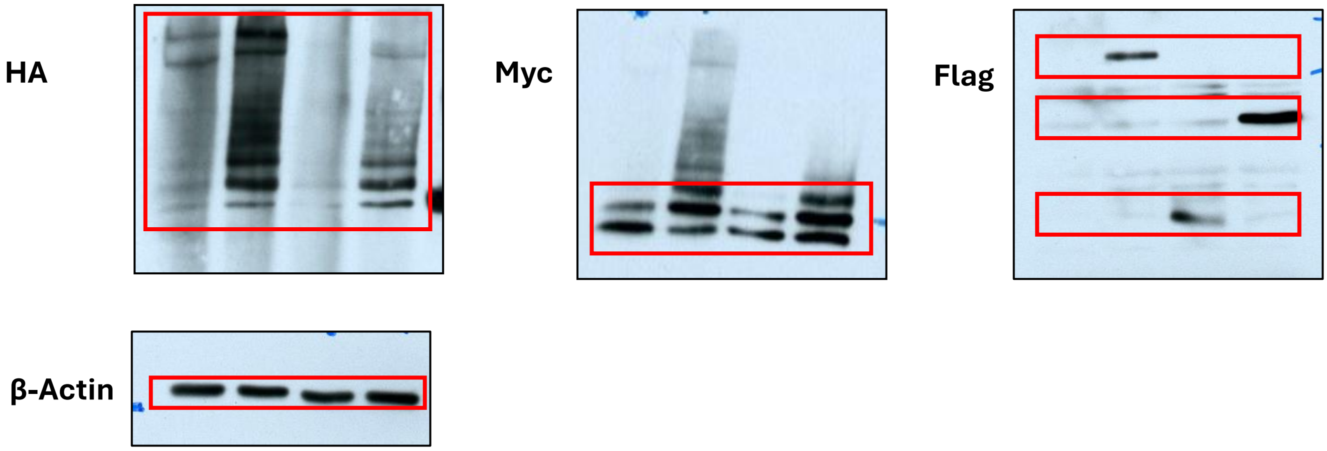

Fig 4E

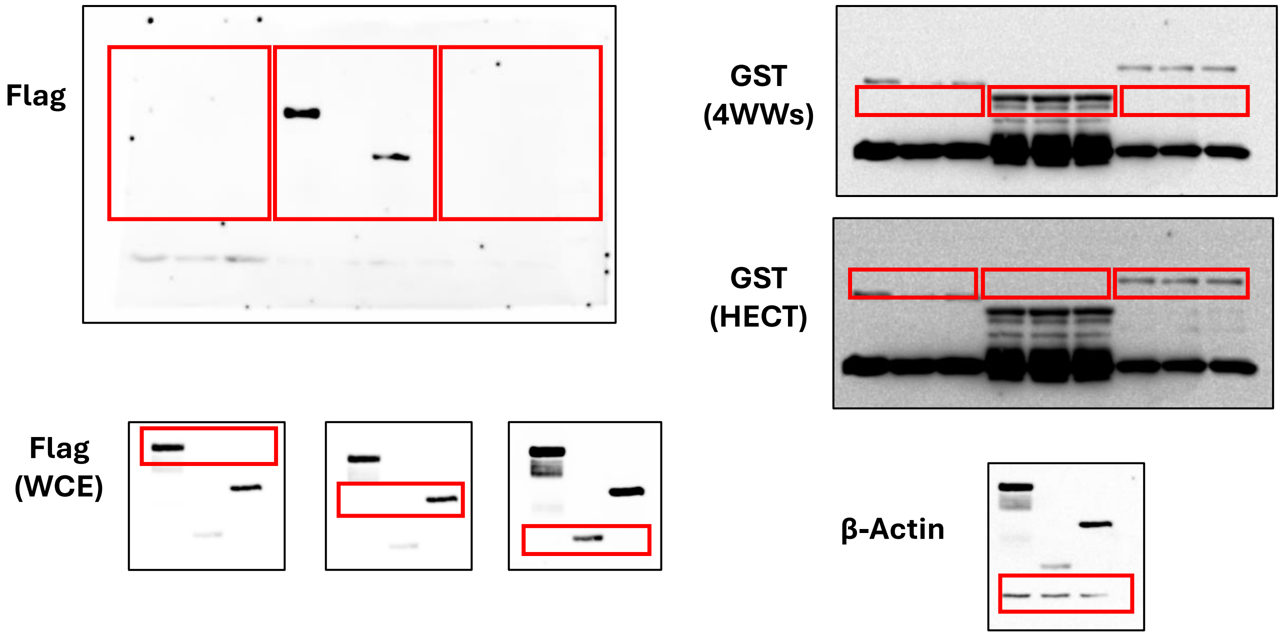

Fig 4G

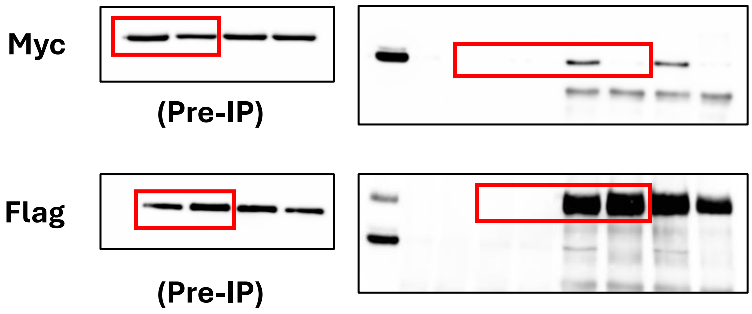

Fig 4H

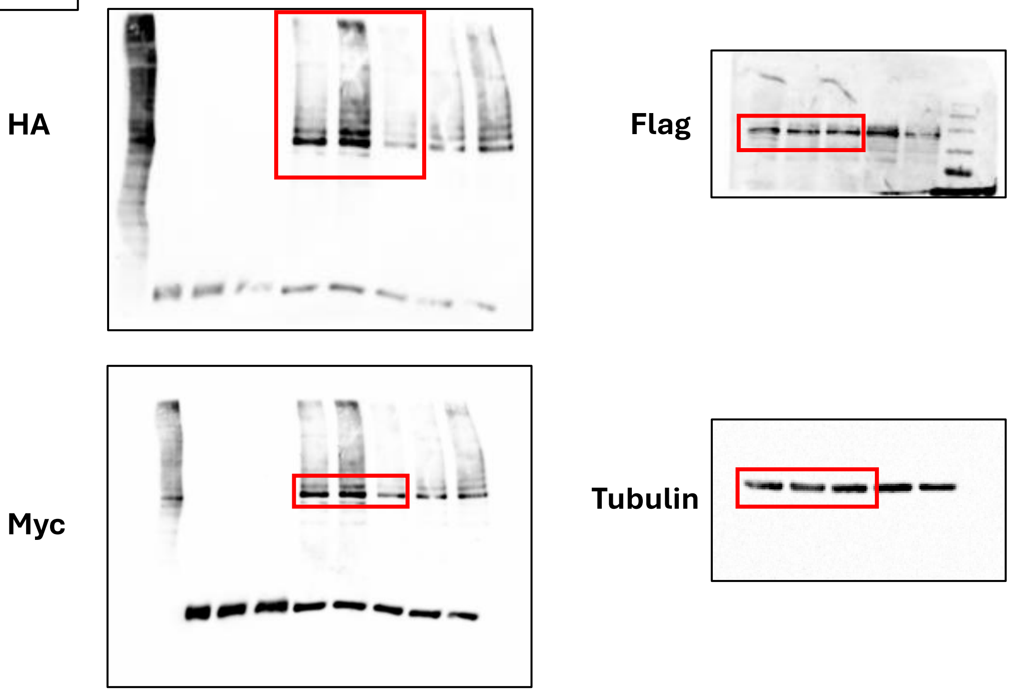

Fig 5D

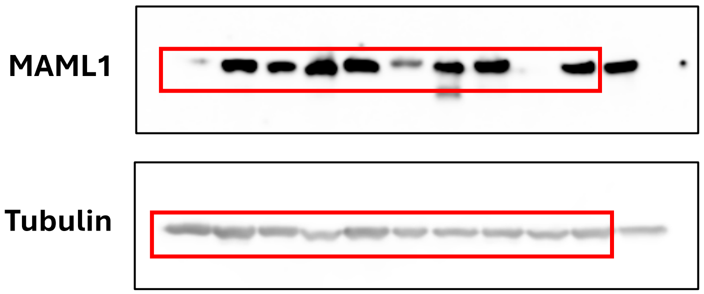

Fig 5E

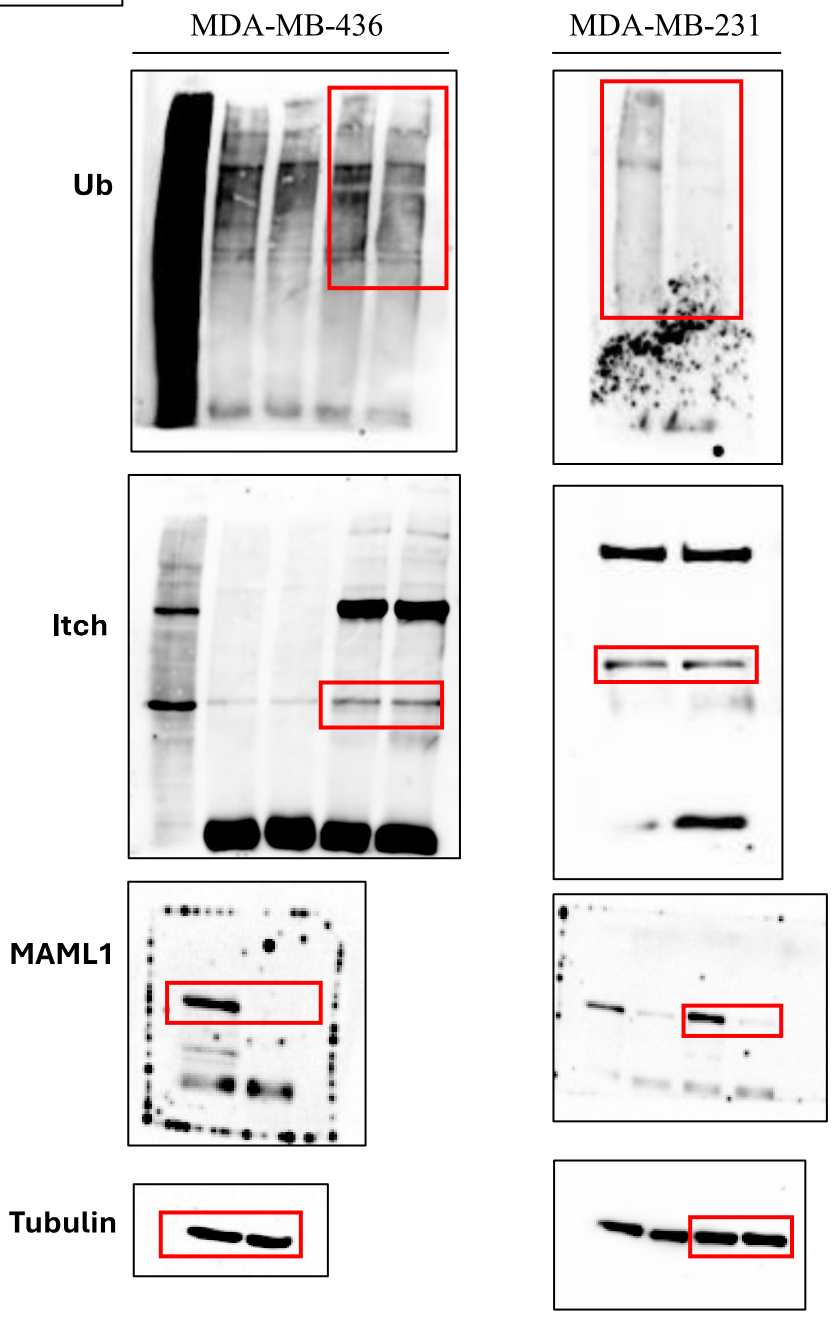

Fig 5G

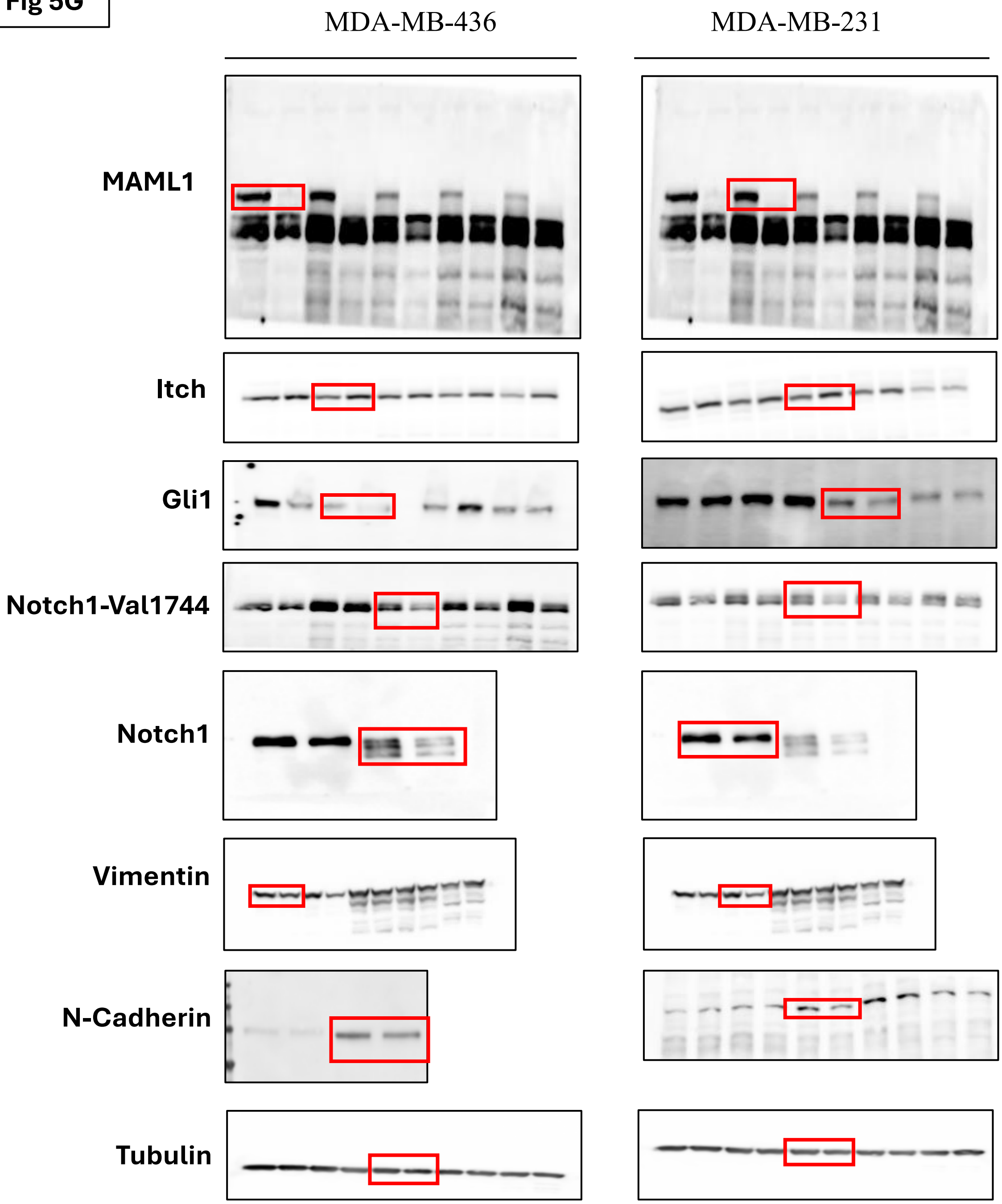

MDA-MB-436
MDA-MB-231
MAML1
Itch
Gli1
Notch1-Val1744
Notch1
Vimentin
N-Cadherin
Tubulin

Fig 6A

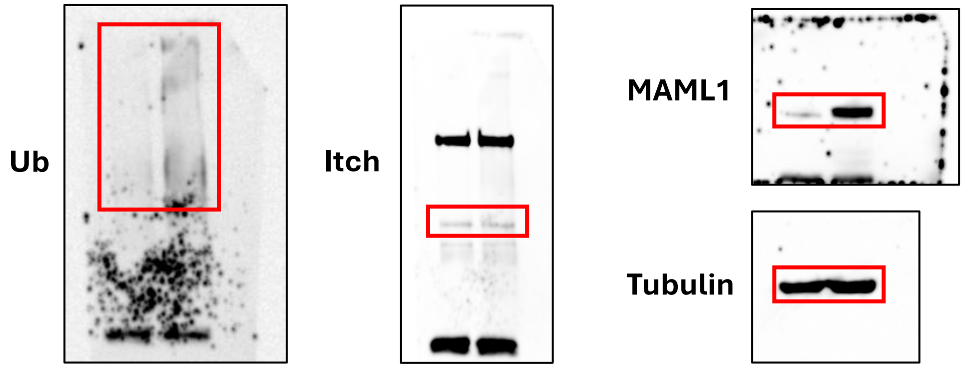

Fig 6D

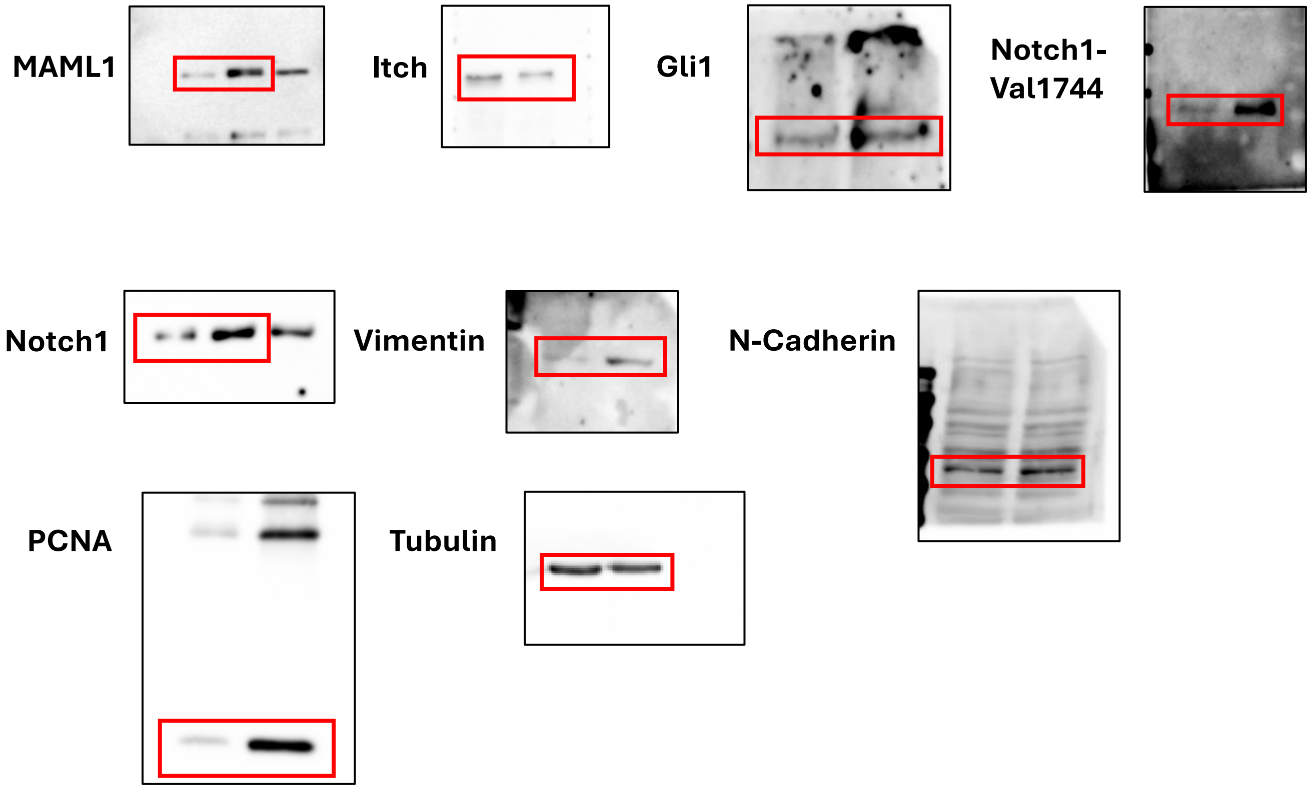

Fig 7A

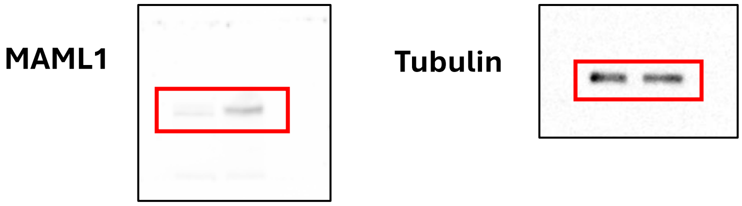

Fig.S3A

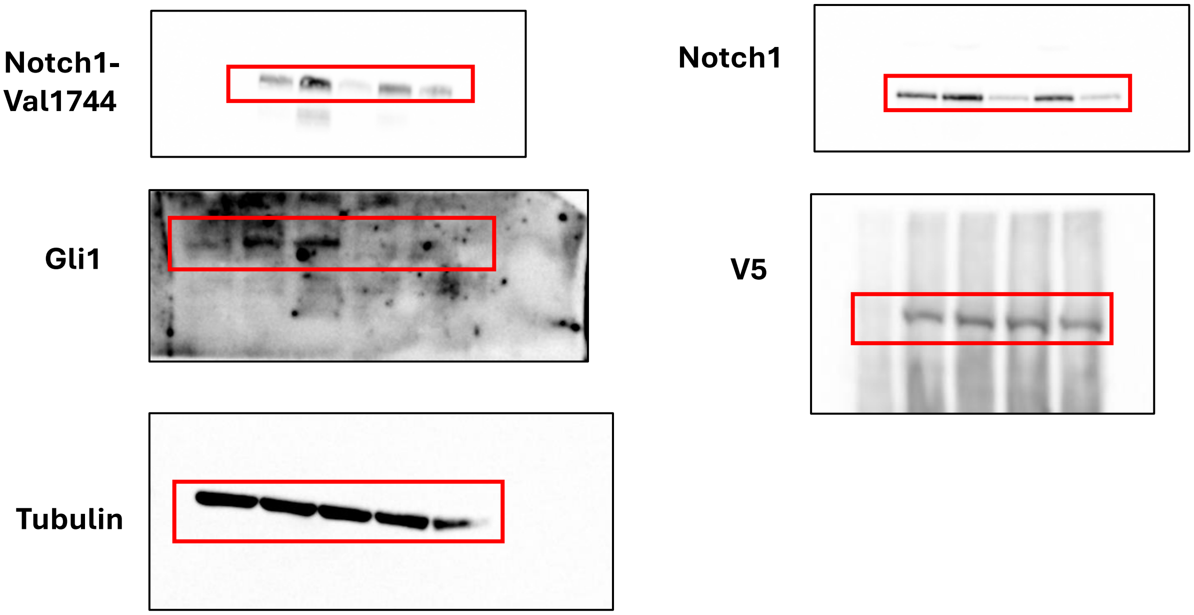

Fig.S3C

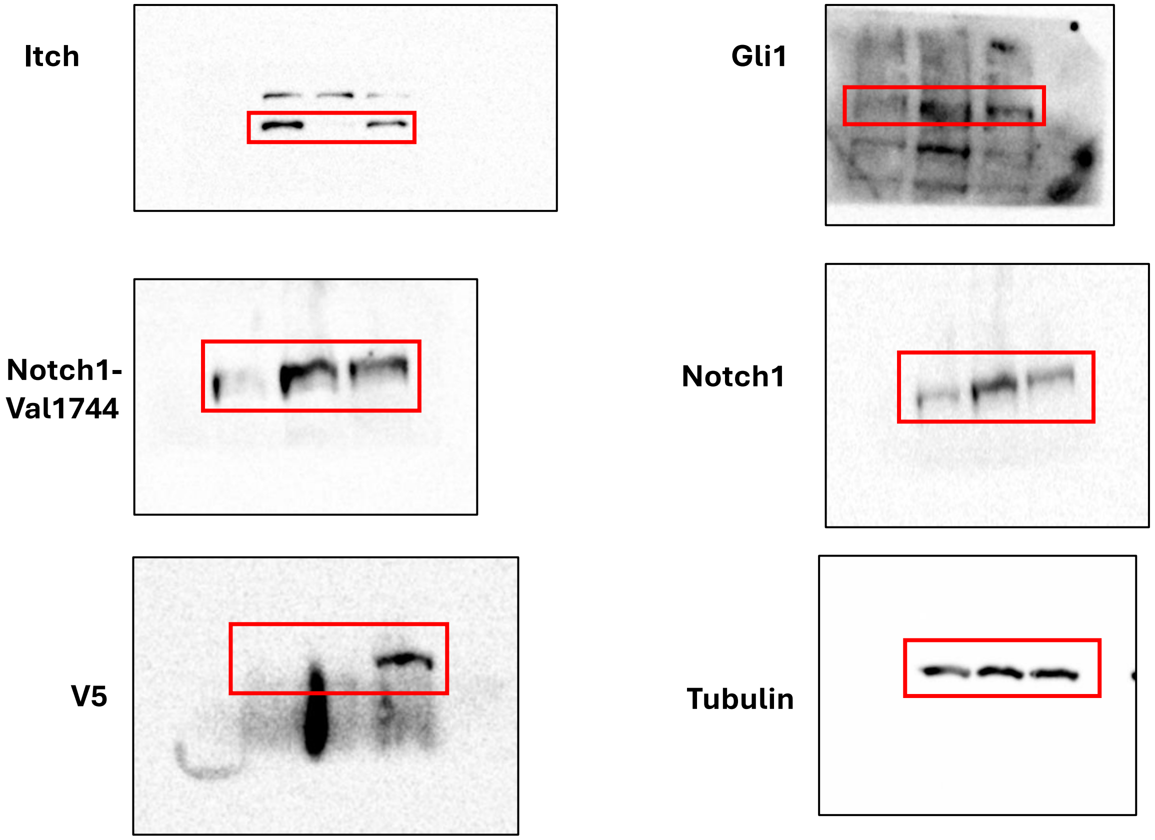

Supplement: Supplementary file 2 — Uncropped Western Blots [file 41418_2025_1613_MOESM2_ESM.pdf]
